# Supplementary material for: Visual mate preference evolution during butterfly speciation is linked to neural processing genes
Source: Nat Commun. 2020 Sep 21;11:4763. doi: 10.1038/s41467-020-18609-z (PMC7506007; doi:10.1038/s41467-020-18609-z)
Supplement: Supplementary file 1 — Supplementary Information [file 41467_2020_18609_MOESM1_ESM.pdf]

# **Supplementary information:** Visual mate preference evolution during butterfly speciation is linked to neural processing genes

Matteo Rossi[1,2], Alexander E. Hausmann[1], Timothy J. Thurman[2,3], Stephen H. Montgomery[4], Riccardo Papa[2,5,6], Chris D. Jiggins[7], W. Owen McMillan[2] & Richard M. Merrill[1,2*]

*1. Division of Evolutionary Biology, LMU, Munich, Germany; 2. Smithsonian Tropical Research Institute, Panama City, Panama; 3. Division of Biological Sciences, University of Montana, USA; 4. School of Biological Sciences, University of Bristol, Bristol, UK; 5. Department of Biology, University of Puerto Rico, San Juan, Puerto Rico; 6. Molecular Sciences and Research Center, University of Puerto Rico, San Juan, Puerto Rico; 7. Department of Zoology, University of Cambridge, Cambridge UK.* [*]corresponding authors**:** rossi@bio.lmu.de, merrill@bio.lmu.de

## Supplementary Methods

**Mapping RNA-seq reads to the *Heliconius cydno* genome.** To determine whether the *H. melpomene* reference genome introduced mapping biases of RNA-seq reads, possibly affecting differential expression estimates, we also mapped to a *H. cydno* assembly/annotation[1]. Generally, we found similar patterns of differential expression when mapping to the two genomes. Since **i)** we observed an equal decrease (~ 40 %) of genes showing 2-fold changes in *melpomene* and *cydno* when mapping to *H. cydno,* at every stage ($P > 0.05$ at every stage, Fisher's Exact test, Table S2A), and **ii)** this decrease was widespread throughout the genome, we concluded that the *H. melpomene* reference genome did not bias differential gene expression analyses. We report the number of reads mapping to both genomes for each adult sample (Table S2B).

**Allele-specific expression in the introgression line.** BC3 hybrids had different combinations of chromosomes segregating for the *melpomene* alleles in a *H. cydno* background. Therefore, in principle, we could not infer *cis*- or *trans*- gene regulatory effects genome-wide from the profiles of allele specific expression (ASE) in these hybrids as for F1 hybrids, due to the diverse trans-acting environments. However, analyses (comparing gene expression levels between hybrids carrying *cyd/melp* vs. *cyd/cyd* regions on chromosomes other than 18) imply that differential expression of the candidate genes seems to be driven by the *H. melpomene* copy difference within the introgressed region on chromosome 18. Therefore, ASE analyses of candidate genes in BC3 hybrids carrying *cyd/melp* alleles on chromosome 18 should indicate whether the differences are due to cis- or trans-regulatory effects from within the introgressed region (Figure S6).

In BC3 hybrids sampled at 156h APF and 60hAPF, the *H. melpomene* and *H. cydno* alleles of the *ionotropic glutamate receptor* (*Grik2*) are expressed at very similar levels ($P > 0.05$ at

both stages, Wald test), suggesting trans-only regulatory effects at these stages for *Grik2*. We detected diffASE expression of *regucalcin2* at 60h APF ($p < 0.05$, Wald test), (and at 156h APF there was a tendency towards up-regulation of the *H. melpomene* allele), but at these stages *regucalcin2* was not detected as differentially expressed between pure species. Thus, although there is evidence for *cis*-regulatory effects for species differences in *regucalcin2* expression during development, the possible effect of *regucalcin2* on behaviour at these stages is less clear.

The region introgressed into *H. cydno* extended ~3.6 Mb beyond the QTL candidate region and seven genes located within this region were differentially expressed (at either stage) in both species and backcross hybrid comparisons (Table S3). We conducted ASE analyses on these seven genes and found evidence for *cis*-regulation for only one (HMEL010030g1, $p < 0.001$, Wald test), which had no annotated function. Unfortunately, we were unable to detect allele-informative reads for the other six genes including a serine protease inhibitor (HMEL014931g1), a CUB domain containing protein (HMEL002560g1), a methyltransferase (HMEL010030g2), a gene with a reverse transcriptase domain (HMEL034294g1), an ionotropic glutamate receptor (HMEL034304g1), and a major facilitator superfamily transporter (HMEL015745g1). (A further two genes on chromosome 18 were located outside of the introgressed region, for which we could not conduct ASE analyses because they were in *cyd*/*cyd* regions).

Our expectation was that *cis*-regulatory elements will normally act on genes in close proximity (the average distance between regulatory elements such as enhancers/repressors and the genes they regulate has been estimated to be less than 100kb[2]), making differentially expressed genes within the QTL peak – and to a lesser extent the candidate region – the best candidates. The QTL might conceivably harbour *cis*-regulatory element(s) acting on these gene(s) at a longer distance on chromosome 18 (*i.e.* outside of the QTL region). Although the closest is at >2.6Mb beyond the QTL peak, we currently cannot completely rule out those

genes, differentially expressed in both species and backcross hybrid comparisons on chromosome 18 (Table S3), as – albeit far less well supported – candidates.

**Supplementary Figures**

**Supplementary Figure 1.** Ternary plots showing the number of 15-minute choice trials in which courtship was initiated towards *melpomene, cydno* or both females for backcross-to-*cydno* males, with different genotypes at the two QTLs retained in our model (on chromosome 1 and chromosome 18). Left ternary axis shows proportion of trials where courtship was initiated towards *H. cydno* female only, bottom axis towards *H. melpomene* female only, and right axis towards both female species. Lines project the three predicted proportions to corresponding values on the three axes and 95% credibility intervals (CrIs) for these proportions are shown as hexagons. Point size is scaled to the number of trials in which the male showed a response and a 'jitter' function has been applied (leading to some dots being jittered to outside the triangle).

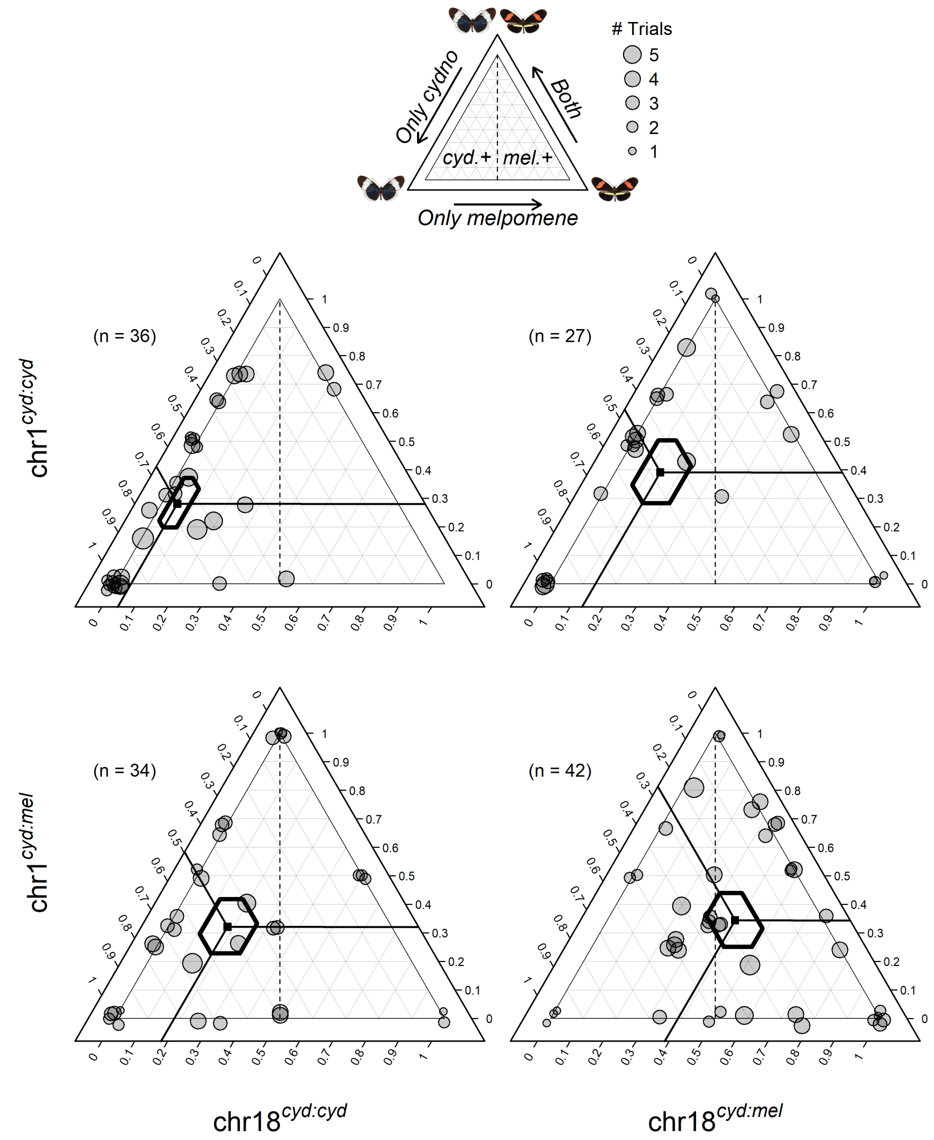

**Supplementary Figure 2.** Results of comparative transcriptomic analyses between *H. melpomene* and *H. cydno* (in the imago) when mapping RNA-seq reads to the *H. melpomene* assembly/annotation (top), and to the *H. cydno* assembly/annotation (bottom), zooming in on the QTL region on chromosome 18. The *x*-axis represents physical position. Points correspond to individual genes, with the *y*-axis indicating the $log_2$(fold-change) for each comparison. The two horizontal dashed lines (at *y*-values of 1 and -1) indicate a 2-fold change in expression. Genes showing a significant 2-fold+ change in expression level between groups are highlighted in orange and blue, where orange indicates higher levels in *melpomene*, blue if in *cydno*. Genes detected as differentially expressed mapping to both *melpomene* and *cydno* genomes are labelled with gene names. dehydr.=2-oxoisovalerate dehydrogenase, OBP=odorant-binding protein.

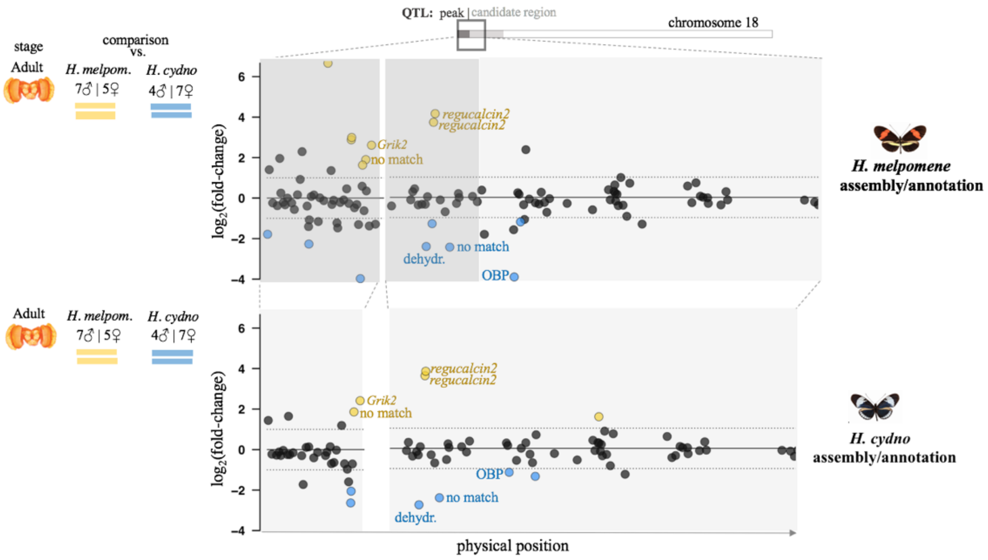

**Supplementary Figure 3.** Crossing design for producing backcross hybrids segregating at the QTL on chromosome 18. This introgression line was created by outcrossing a male hybrid to *H. cydno* females over three generations, selecting a hybrid male that showed a red band on the wing at each generation. This meant that these males carried one copy of the *H. melpomene* allele at the *optix* locus. We expected that, following recombination (which occurs in males), by the fourth generation we would remain with two types of individuals: either *cyd*/*melp* or *cyd*/*cyd* at the level of the *optix* region (which approximately corresponds to the region associated with male preference behaviour).

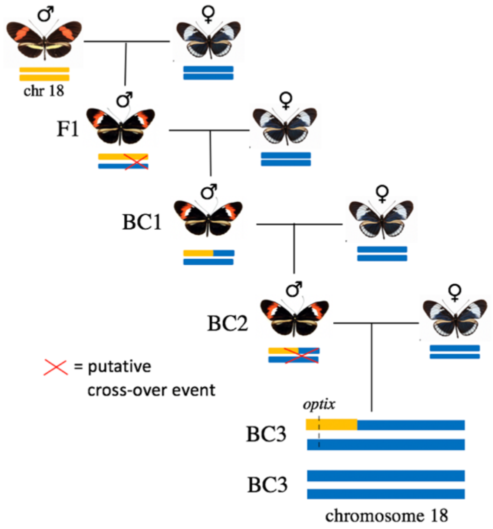

**Supplementary Figure 4.** Differential gene expression at the QTL region at pupal stages. Left: summary of the comparative transcriptomic analyses with stage, number of samples and chromosome 18 composition. Right: the corresponding results, zooming in on the QTL region on chromosome 18. The *x*-axis represents physical position. The QTL peak, and the rest of the QTL 1.5 LOD candidate region are shown in green and purple, respectively. Points correspond to individual genes, with the *y*-axis indicating the $log_2$(fold-change) for each comparison. The two horizontal dashed lines (at *y*-values of 1 and -1) indicate a 2-fold change in expression. Genes showing a significant 2-fold+ change in expression level between groups are highlighted in orange and blue, where orange indicates higher levels in *melpomene* or in the hybrids *cyd*/*melp* (blue if in *cydno* – hybrids *cyd*/*cyd*). Vertical dashed lines highlight those genes that are differentially expressed between *H. melpomene* and *H. cydno* AND between *cyd/melp* vs *cyd/cyd* individuals, at the same stage. One gene highlighted by a dashed fuchsia vertical line was excluded because it showed reversal of the fold change when mapping RNA-seq reads to the *H. cydno* genome.

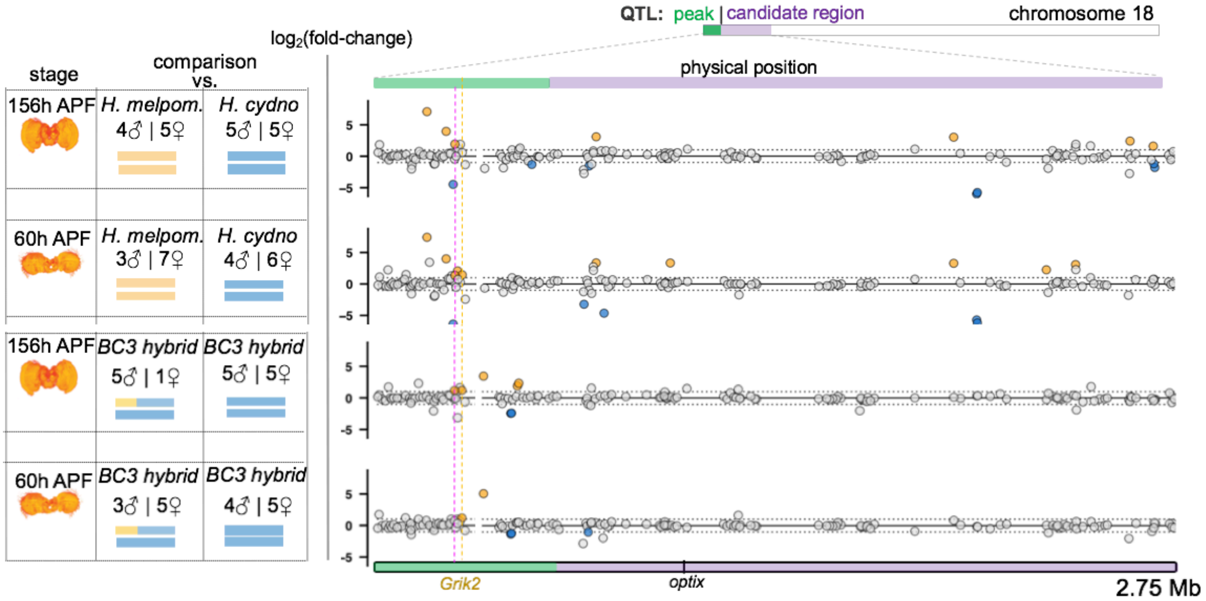

**Supplementary Figure 5.** A) Schematic representation of hybrid pupae (sampled at 156h APF) genome composition. Columns represent chromosomes, rows represent individuals, orange indicates *cyd/melp* regions, blue indicates *cyd/cyd* regions. B) Genome composition of (a subset of) BC3 hybrids. We calculated the fraction of SNPs and indels that each BC3 hybrid, one *cydno* and one F1 hybrid samples shared with *H. melpomene* and *H. cydno* samples, in non-overlapping 100kb windows. x-axes represent physical position (for each chromosome), y-axes fractions of shared variants with *melpomene* (in gold) and with *cydno* (in light blue). Matching variant fractions between BC3 hybrids and the F1 hybrid, indicating heterozygous regions, are highlighted with a gold bar underneath. Note that the general trend of higher number of variants shared with *H. cydno* in heterozygous regions is due to the fact that we inferred variants by mapping to the *H. melpomene* genome (and used variant sites only for this analysis).

**A)**

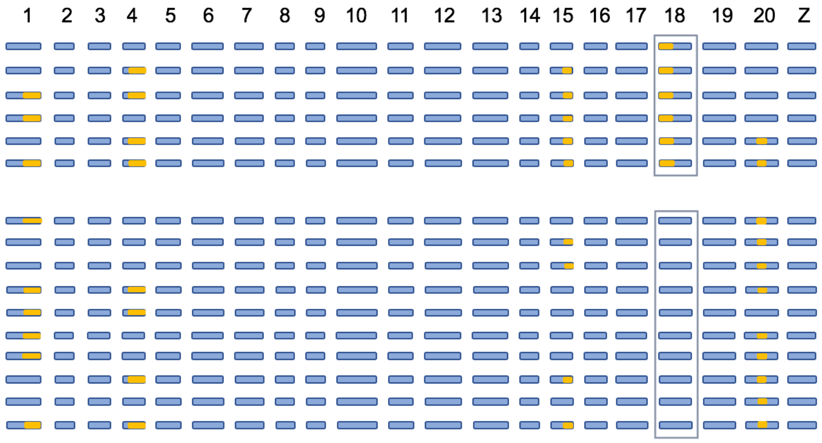


**B)**

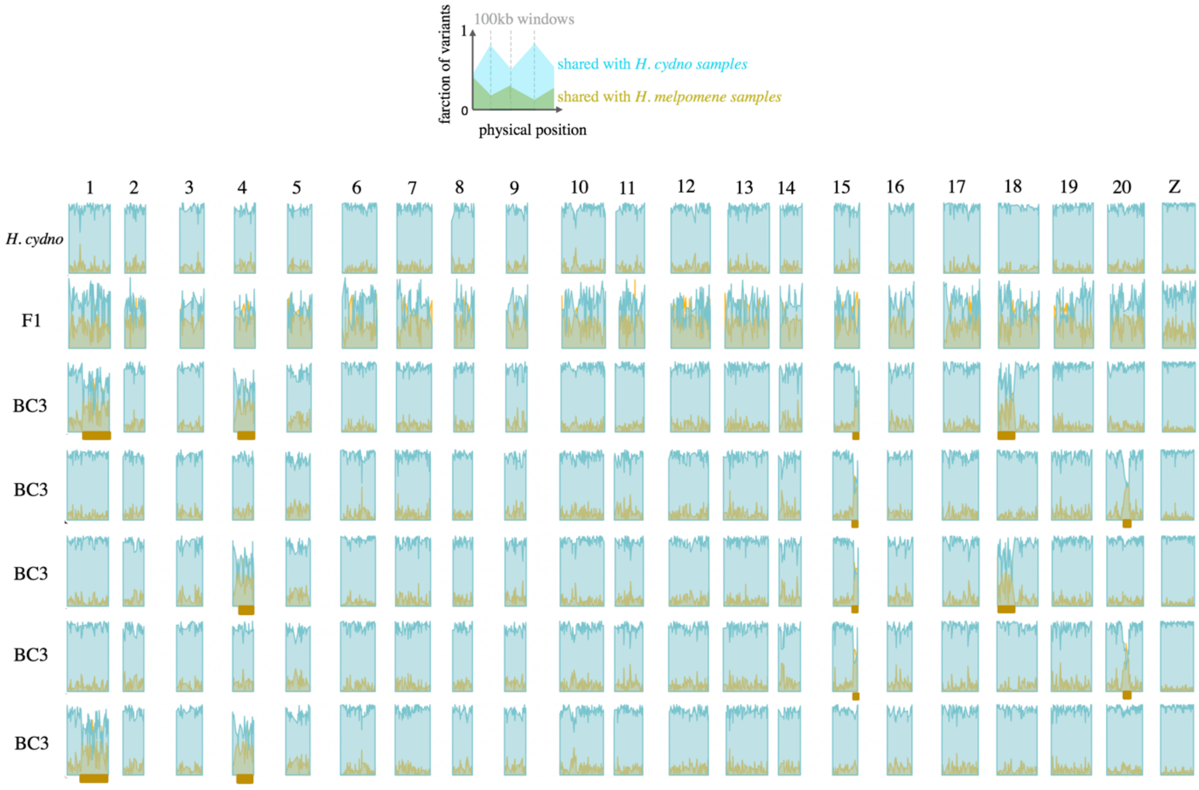

**Supplementary Figure 6.** Allele specific expression profiles of candidate genes at pupal stages. Points indicate the mean value, and bars the standard error, of the (base 2) logarithmic fold change in expression between parental species (vertical) (n=9 (156h APF) and n=10 (60h APF) biologically independent samples) and the alleles in BC3 hybrids *cyd/melp* at the QTL (horizontal) (n=6 (156h APF) and n=8 (60h APF) biologically independent samples), for candidate genes (as defined in the transcript-guided annotation). Dashed lines indicate the threshold for a 2-fold change in expression for the genes in the species (horizontal), and for the alleles in the hybrids (vertical).

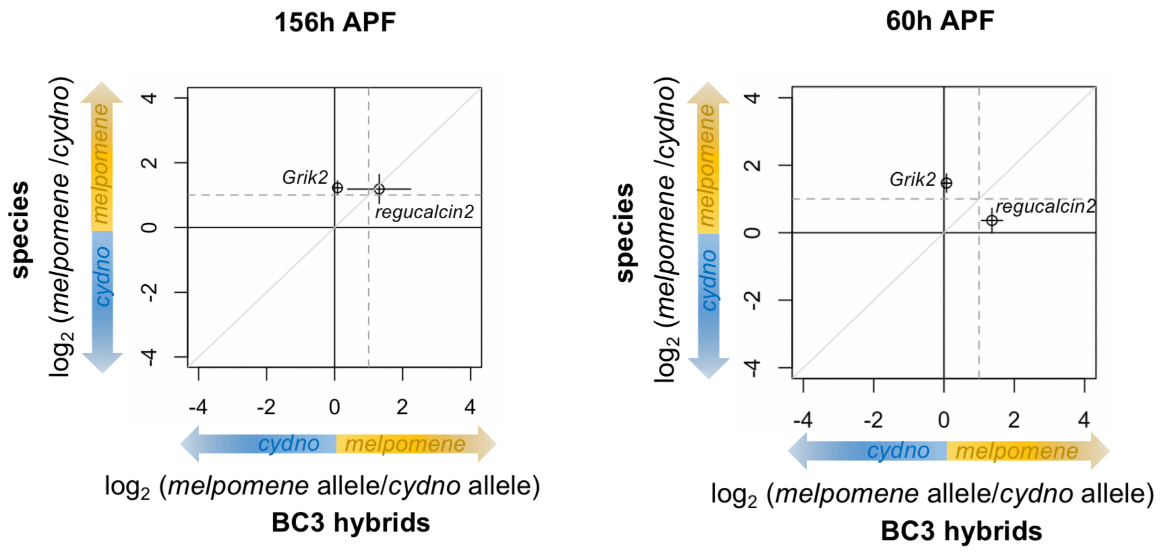


**Supplementary Figure 7.** Distribution of the (base 2) logarithmic fold change in allele expression. Coloured bars indicate the number of genes showing a bias in expression for the *H. cydno* allele (in blue) and for the *H. melpomene* allele (in yellow). Values departing from 0 on the x-axis, indicate an increase in the fold change for the *H. cydno* allele (negative values) or for the *H. melpomene* allele (positive values), respectively.

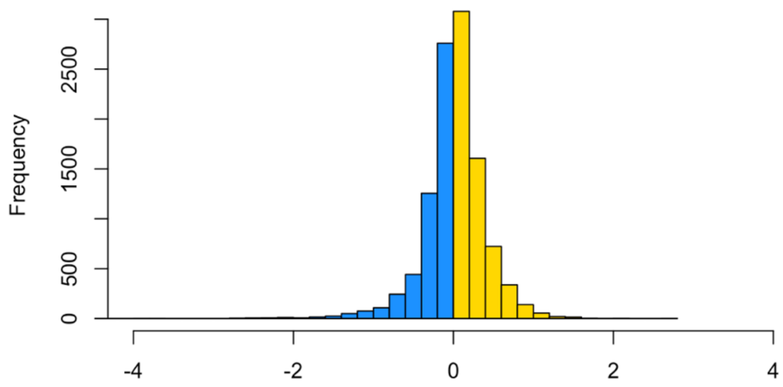

## Supplementary Tables

**Supplementary Table 1.** List of differentially expressed genes in species and hybrids comparisons.

**A)** QTL chromosome 1. Orange indicates genes up-regulated in *H. melpomene*, and blue those up-regulated in *H. cydno.* Those genes found to be differentially expressed when also mapping to the *H. cydno* genome are highlighted in bold.

| # | Gene name (Hmel2.5) | Annotated function | Species comparison | | | F1 hybrid vs. *H. cydno* |
|---|---|---|---|---|---|---|
| | | | 60h APF | 156h APF | Adult | Adult |
| 1 | HMEL002973g1 | No match | ✓ | ✓ | ✓ | ✓ |
| **2** | **HMEL003796g1** | **Regulation of enolase protein 1** | ✓ | ✗ | ✓ | ✓ |
| **3** | **HMEL011272g1** | **no match** | ✗ | ✓ | ✓ | ✗ |
| 4 | HMEL030024g1 | Ribonuclease H superfamily | ✓ | ✓ | ✓ | ✗ |
| 5 | HMEL030042g1 | SWR1-complex protein 5 | ✗ | ✗ | ✓ | ✗ |
| 6 | HMEL030052g1 | reverse transcriptase | ✓ | ✓ | ✓ | ✗ |
| 7 | HMEL005260g1 | unknown | ✗ | ✓ | ✗ | ✗ |
| 8 | HMEL030040g1 | No match | ✗ | ✓ | ✗ | ✗ |
| 9 | HMEL030037g1 | No match | ✓ | ✓ | ✗ | ✗ |
| 10 | HMEL010076g1 | Amino acid transporter | ✓ | ✗ | ✗ | ✗ |

**B**) QTL chromosome 18. Those genes found to be differentially expressed when also mapping to the *H. cydno* genome are highlighted in bold. Genes annotated as distinct but sharing the same number in the table (#) were later found to be single genes (see second paragraph of the Results section).

| # | Gene name (Hmel2.5) | Annotated function | Species comparison | | | Hybrids comparison | | |
|---|---|---|---|---|---|---|---|---|
| | | | 60h APF | 156h APF | Adult | 60h APF | 156h APF | Adult |
| **1** | **HMEL009992g1** | **No match** | ✓ | ✗ | ✓ | ✗ | ✗ | ✓ |
| **1** | **HMEL009992g4** | ***Ionotropic glutamate receptor*** | ✓ | ✗ | ✓ | ✓ | ✓ | ✓ |
| **2** | **HMEL009996g1** | ***Gag-related protein*** | ✓ | ✓ | ✓ | ✗ | ✗ | ✗ |
| 3 | HMEL034168g1 | unknown | ✗ | ✗ | ✓ | ✗ | ✗ | ✗ |
| 4 | HMEL034173g1 | *SWR1-complex protein 5* | ✗ | ✗ | ✓ | ✗ | ✗ | ✗ |
| 5 | HMEL034176g1 | *Aspartic peptidase* | ✓ | ✓ | ✓ | ✗ | ✗ | ✗ |
| 6 | HMEL034184g1 | No match | ✗ | ✗ | ✓ | ✗ | ✗ | ✗ |
| **7** | **HMEL034185g1** | **No match** | ✓ | ✓ | ✓ | ✗ | ✗ | ✗ |
| 8 | HMEL034187g1 | *Major facilitator superfamily (MFS) transporter* | ✓ | ✓ | ✓ | ✗ | ✓ | ✓ |
| **9** | **HMEL003176** | ***Odorant binding protein*** | ✗ | ✓ | ✓ | ✓ | ✗ | ✗ |
| 10 | HMEL013551g1 | *2-oxoisovalerate dehydrogenase* | ✗ | ✗ | ✗ | ✓ | ✓ | ✗ |
| **10** | **HMEL013551g2** | ***2-oxoisovalerate dehydrogenase*** | ✗ | ✗ | ✓ | ✓ | ✗ | ✗ |
| 11 | HMEL013551g4 | *SMP-30/regucalcin* | ✗ | ✗ | ✓ | ✗ | ✗ | ✗ |
| **12** | **HMEL013552g1** | ***SMP-30/regucalcin*** | ✗ | ✗ | ✓ | ✗ | ✓ | ✓ |
| **12** | **HMEL034199g1** | ***SMP-30/regucalcin*** | ✗ | ✗ | ✓ | ✗ | ✓ | ✓ |
| 13 | HMEL014202g1 | *Catalase* | ✗ | ✗ | ✓ | ✗ | ✗ | ✗ |
| **14** | **HMEL014202g3** | ***Catalase*** | ✗ | ✓ | ✗ | ✗ | ✗ | ✗ |
| **15** | **HMEL034201g1** | ***No match*** | ✗ | ✗ | ✓ | ✗ | ✗ | ✗ |
| **16** | **HMEL034205g1** | ***No match*** | ✓ | ✗ | ✓ | ✗ | ✗ | ✗ |
| 17 | HMEL034227g1 | *Ribonuclease H superfamily* | ✓ | ✓ | ✓ | ✗ | ✗ | ✗ |
| 18 | HMEL034229g1 | *Endonuclease/exonuclease/phosphatase superfamily* | ✓ | ✓ | ✓ | ✗ | ✗ | ✓ |
| 19 | HMEL034230g1 | *No match* | ✓ | ✓ | ✓ | ✗ | ✗ | ✗ |
| **20** | **HMEL003863g1** | ***Vacuolar protein sorting-associated (VPS) protein*** | ✓ | ✓ | ✗ | ✗ | ✗ | ✗ |
| 21 | HMEL003863g3 | *No match* | ✓ | ✗ | ✗ | ✗ | ✗ | ✗ |
| **22** | **HMEL006662g1** | ***Serpin family protein*** | ✗ | ✓ | ✗ | ✗ | ✗ | ✗ |
| **23** | **HMEL006663** | ***Odorant binding protein*** | ✗ | ✓ | ✗ | ✗ | ✗ | ✗ |
| **24** | **HMEL022553** | ***Odorant binding protein*** | ✗ | ✓ | ✗ | ✗ | ✗ | ✗ |
| 25 | HMEL001038g1 | *Monocarboxylate transporter* | ✓ | ✗ | ✗ | ✗ | ✗ | ✗ |
| 26 | HMEL014190g1 | *unknown function* | ✓ | ✗ | ✗ | ✗ | ✗ | ✗ |
| 27 | HMEL034236g1 | *No match* | ✓ | ✗ | ✗ | ✗ | ✗ | ✗ |
| 28 | HMEL034189g1 | *PiggyBac transposable element-derived protein* | ✗ | ✗ | ✗ | ✗ | ✗ | ✓ |
| 29 | HMEL034246g1 | *No match* | ✗ | ✗ | ✗ | ✗ | ✗ | ✓ |
| 30 | HMEL034195g1 | *Gag-related protein* | ✗ | ✗ | ✗ | ✓ | ✓ | ✗ |

**Supplementary Table 2.**

**A)** Number of genes showing significant >2-fold change in expression, at different stages, mapping to the *H. melpomene* and to the *H. cydno* genomes. Note that the considerable reduction in the number of genes detected as differentially expressed when mapping to *H. cydno* is most likely a result of the lower quality/completeness of the *H. cydno* genome assembly.

| Stage | Mapping to: | Up-regulated in *H. melpomene* | Up-regulated in *H. cydno* |
|---|---|---|---|
| Adult | *H. melpomene* | 694 | 733 |
| | *H. cydno* | 390 | 451 |
| 156h APF | *H. melpomene* | 837 | 667 |
| | *H. cydno* | 518 | 403 |
| 60h APF | *H. melpomene* | 846 | 642 |
| | *H. cydno* | 490 | 376 |

**B)** Number of reads mapping to the *H. melpomene* and the *H. cydno* genome for every species sample at the adult stage.

| ***H. melpomene*** | | | ***H. cydno*** | | |
|---|---|---|---|---|---|
| **ID** | **mapped to *H. mel*** | **mapped to *H. cyd*** | **ID** | **mapped to *H. mel*** | **mapped to *H. cyd*** |
| **45** | 14660303 | 14399385 | **50** | 17617312 | 10276127 |
| **47** | 7157775 | 7106443 | **51** | 14965350 | 11157953 |
| **53** | 14013806 | 13804267 | **57** | 17460091 | 10958479 |
| **70** | 11850181 | 11656033 | **58** | 20474961 | 14651415 |
| **71** | 11218116 | 11023267 | **67** | 27415308 | 15742957 |
| **78** | 14114255 | 13618292 | **68** | 22159665 | 12175286 |
| **80** | 14551439 | 14097670 | **81** | 34947798 | 19085351 |
| **83** | 13616160 | 13260160 | **82** | 15525206 | 10264627 |
| **100** | 13771837 | 13458527 | **84** | 13139892 | 9836625 |
| **104** | 12802894 | 12664628 | **98** | 18846622 | 12585701 |
| **128** | 13840154 | 13489851 | **99** | 20309368 | 13271219 |
| **218** | 16942305 | 16812556 | | | |

**Supplementary Table 3.** List of differentially expressed genes in both species and backcross hybrid comparisons (at 60h APF or 156h APF). Genes annotated as distinct but sharing the same number in the table (#) were later found to be single genes. *gene excluded because it showed reversal of the fold change when mapping to the *H. cydno* genome.

| # | Gene name (Hmel2.5) | Annotated function | 60h APF | 156h APF | chromosome | Start (bp) position | End (bp) position |
|---|---|---|---|---|---|---|---|
| 1 | HMEL009992g4 | *Ionotropic glutamate receptor* | ✓ | ✗ | 18 | 334856 | 345144 |
| * | HMEL034187g1 | *no match* | ✗ | ✓ | 18 | 314734 | 315069 |
| 2 | HMEL014931g1 | Serine endopeptidase inhibitor | ✓ | ✓ | 18 | 3260635 | 3264491 |
| 3 | HMEL034294g1 | Reverse transcriptase domain | ✓ | ✓ | 18 | 4369133 | 4370180 |
| 4 | HMEL002560g1 | CUB domain | ✓ | ✗ | 18 | 4679193 | 4684506 |
| 5 | HMEL034304g1 | *Ionotropic glutamate receptor* | ✓ | ✗ | 18 | 4935938 | 4944768 |
| 6 | HMEL010030g2 | *Methyltransferase* | ✓ | ✗ | 18 | 5503482 | 5505157 |
| 6 | HMEL010030g3 | no match | ✓ | ✗ | 18 | 5506399 | 5508526 |
| 7 | HMEL010030g1 | no match | ✓ | ✓ | 18 | 5496842 | 5498456 |
| 8 | HMEL015745g1 | *Major facilitator superfamily* | ✗ | ✓ | 18 | 5976551 | 5982484 |
| 9 | HMEL014795g1 | *Major facilitator superfamily* | ✗ | ✓ | 18 | 10194935 | 10200873 |
| 10 | HMEL015842g1 | *Alpha crystallin/heat shock protein* | ✗ | ✓ | 18 | 13546671 | 13547216 |
| 11 | HMEL030024g1 | *Ribonuclease H superfamily* | ✗ | ✓ | 1 | 1009751 | 1013056 |

**Supplementary Table 4.** Heterozygosity on the *Z*-chromosome. Heterozygosity is calculated as proportion of variants (SNPs and indels) which are heterozygous, in each sample, rounded at the second decimal place (note that variant sites were inferred having mapped to the *H. melpomene* genome).

| ***H. melpomene*** | | | | ***H. cydno*** | | | |
|---|---|---|---|---|---|---|---|
| Males | | Females | | Males | | Females | |
| **ID** | Het. | **ID** | Het. | **ID** | Het. | **ID** | Het. |
| Adults | | | | | | | |
| **45** | 0.49 | **53** | 0.04 | **57** | 0.23 | **50** | 0.02 |
| **47** | 0.46 | **78** | 0.05 | **82** | 0.23 | **51** | 0.02 |
| **70** | 0.47 | **80** | 0.04 | **98** | 0.25 | **58** | 0.02 |
| **71** | 0.48 | **128** | 0.04 | **99** | 0.24 | **67** | 0.02 |
| **83** | 0.46 | **218** | 0.05 | | | **68** | 0.01 |
| **100** | 0.49 | | | | | **81** | 0.02 |
| **104** | 0.46 | | | | | **84** | 0.02 |
| 156h APF | | | | | | | |
| **5** | 0.47 | **6** | 0.05 | **4** | 0.26 | **13** | 0.02 |
| **14** | 0.49 | **18** | 0.05 | **8** | 0.26 | **21** | 0.04 |
| **17** | 0.49 | **24** | 0.05 | **142** | 0.30 | **30** | 0.03 |
| **184** | 0.50 | **150** | 0.05 | **151** | 0.29 | **137** | 0.04 |
| | | **220** | 0.06 | **156** | 0.29 | **168** | 0.04 |
| 60h APF | | | | | | | |
| **92** | 0.48 | **87** | 0.06 | **85** | 0.26 | **90** | 0.02 |
| **97** | 0.49 | **95** | 0.05 | **86** | 0.27 | **118** | 0.02 |
| **115** | 0.47 | **117** | 0.05 | **119** | 0.27 | **125** | 0.02 |
| | | **124** | 0.04 | **146** | 0.26 | **144** | 0.02 |
| | | **149** | 0.06 | | | **162** | 0.02 |
| | | **164** | 0.05 | | | **200** | 0.02 |
| | | **208** | 0.06 | | | | |

| **Hybrids** | | | | | | | |
|---|---|---|---|---|---|---|---|
| Males | | Females | | Males | | Females | |
| **ID** | Het. | **ID** | Het. | **ID** | Het. | **ID** | Het. |
| **F1 hybrids (adults)** | | | | | | | |
| **42** | 0.64 | **56** | 0.05 | | | | |
| **49** | 0.64 | **69** | 0.04 | | | | |
| **Introgression line -156h APF** | | | | **Introgression line – 60h APF** | | | |
| **105** | 0.26 | **108** | 0.02 | **152** | 0.27 | **161** | 0.02 |
| **116** | 0.27 | **123** | 0.02 | **193** | 0.27 | **165** | 0.02 |
| **126** | 0.27 | **136** | 0.02 | **198** | 0.25 | **179** | 0.02 |
| **131** | 0.27 | **139** | 0.02 | **199** | 0.27 | **187** | 0.02 |
| **133** | 0.26 | **140** | 0.02 | **201** | 0.26 | **188** | 0.02 |
| **154** | 0.26 | **166** | 0.02 | **212** | 0.28 | **192** | 0.02 |
| **155** | 0.26 | | | **215** | 0.27 | **197** | 0.02 |
| **183** | 0.27 | | | | | **209** | 0.03 |
| **185** | 0.27 | | | | | **214** | 0.02 |
| **189** | 0.27 | | | | | **224** | 0.02 |

**Supplementary References**


1. Darragh, K. *et al.* A novel terpene synthase produces an anti-aphrodisiac pheromone in the butterfly *Heliconius melpomene*. Preprint at https://www.biorxiv.org/content/10.1101/779678v1 (2019).
2. Gaffney, D. J. Mapping and predicting gene–enhancer interactions. *Nat. Gen.* ***51***, 1662-1663 (2019).
