# Supplementary material for: Visual mate preference evolution during butterfly speciation is linked to neural processing genes
Source: Nat Commun. 2020 Sep 21;11:4763. doi: 10.1038/s41467-020-18609-z (PMC7506007; doi:10.1038/s41467-020-18609-z)
Supplement: Supplementary file 3 — Reporting Summary [file 41467_2020_18609_MOESM3_ESM.pdf]

nature research

Corresponding author(s): Richard Merrill

Last updated by author(s): Aug 25, 2020

Reporting Summary

Nature Research wishes to improve the reproducibility of the work that we publish. This form provides structure for consistency and transparency in reporting. For further information on Nature Research policies, see our Editorial Policies and the Editorial Policy Checklist.

Statistics

For all statistical analyses, confirm that the following items are present in the figure legend, table legend, main text, or Methods section.

n/a	Confirmed	
☐	☒	The exact sample size (*n*) for each experimental group/condition, given as a discrete number and unit of measurement
☐	☒	A statement on whether measurements were taken from distinct samples or whether the same sample was measured repeatedly
☐	☒	The statistical test(s) used AND whether they are one- or two-sided<br>*Only common tests should be described solely by name; describe more complex techniques in the Methods section.*
☐	☒	A description of all covariates tested
☐	☒	A description of any assumptions or corrections, such as tests of normality and adjustment for multiple comparisons
☐	☒	A full description of the statistical parameters including central tendency (e.g. means) or other basic estimates (e.g. regression coefficient) AND variation (e.g. standard deviation) or associated estimates of uncertainty (e.g. confidence intervals)
☐	☒	For null hypothesis testing, the test statistic (e.g. *F*, *t*, *r*) with confidence intervals, effect sizes, degrees of freedom and *P* value noted<br>*Give P values as exact values whenever suitable.*
☐	☒	For Bayesian analysis, information on the choice of priors and Markov chain Monte Carlo settings
☐	☒	For hierarchical and complex designs, identification of the appropriate level for tests and full reporting of outcomes
☒	☐	Estimates of effect sizes (e.g. Cohen's *d*, Pearson's *r*), indicating how they were calculated

Our web collection on statistics for biologists contains articles on many of the points above.

Software and code

Policy information about availability of computer code

Data collection	No software was used for data collection
Data analysis	Analysis scripts are available at: https://github.com/SpeciationBehaviour/neural_genes_heliconius.git<br>Statistical analyses: R v.4.0<br>Quality control: FastQC v.0.11.4<br>Trimming: TrimGalore v.0.4.4<br>Mapping: STAR v.2.4.2a<br>Mapping count: HTseq v.0.9.1<br>Differential expression: DESeq2 v.1.20<br>Annotate function: InterProScan v.5<br>Enrichment test: PANTHER v.15.0<br>Transcript-based annotation: Cufflinks v.2.2.0<br>Marking duplicates: Picard v.1.8.0<br>Variant calling: GATK v.3.8<br>Handle mapping files: bcftools v.1.4.1, samtools v.1.4.1, vcftools v.0.1.14<br>Predict SNPs effects: SNPEff v.4.3<br>Predict effect on protein function: PROVEAN v.1.1.3

For manuscripts utilizing custom algorithms or software that are central to the research but not yet described in published literature, software must be made available to editors and reviewers. We strongly encourage code deposition in a community repository (e.g. GitHub). See the Nature Research guidelines for submitting code & software for further information.

Data

Policy information about availability of data

All manuscripts must include a data availability statement. This statement should provide the following information, where applicable:

- Accession codes, unique identifiers, or web links for publicly available datasets
- A list of figures that have associated raw data
- A description of any restrictions on data availability

RNA-seq data (raw reads) have been deposited in the European Nucleotide Archive (ENA) (accession number: PRJEB39935) and are available at [http://www.ebi.ac.uk/ena/data/view/PRJEB39935]. Individual samples' accession numbers and detailed metadata are reported in the Supplementary Data 1. Previously published genomic data are available at: [https://www.ebi.ac.uk/ena/browser/view/PRJEB1749] and at [https://www.ncbi.nlm.nih.gov/bioproject/PRJEB11772].

Field-specific reporting

Please select the one below that is the best fit for your research. If you are not sure, read the appropriate sections before making your selection.

☒ Life sciences ☐ Behavioural & social sciences ☐ Ecological, evolutionary & environmental sciences

For a reference copy of the document with all sections, see nature.com/documents/nr-reporting-summary-flat.pdf

Life sciences study design

All studies must disclose on these points even when the disclosure is negative.

Sample size	Behavioral data: 146 backcross males for which we had genotype data. RNAseq-data: 36 H. melpomene individuals, 36 H. cydno individuals, 10 first-generation hybrid individuals, 33 third-generation backcross-hybrid individuals. Data for behavioral analysis were acquired from a previous analysis, which were themselves determined by the maximum number of individuals that could be phenotyped across a two year 'field season'. Sample for RNAseq were dependent on the number of individuals that could be raised, and sequenced, but are favourably comparable to similar analyses of gene expression differences.
Data exclusions	Behavioral data: males that did not initiate courtship to any female across trials were excluded from analyses because they had no phenotype to associate with their genotype, resulting in a dataset of 139 males.
Replication	Behavioral data: whenever possible male choice trials were repeated for each male (median = 5 trials). Comparative transcriptomic analyses for 'pure' individuals and F1 hybrids, at the adult stage, were repeated across two independent sample collections, as described in the manuscript.
Randomization	RNA-seq: to avoid lane effects the distribution of the species and hybrid samples was randomized on the sequencing platform.
Blinding	Behavioural analysis involved reanalysis of previously published data (Merrill et al PLoS Biology 2019), as stated in this previously published paper manuscript, investigators were blind to individual genotypes, except at Mendelian colour wing pattern loci

Reporting for specific materials, systems and methods

We require information from authors about some types of materials, experimental systems and methods used in many studies. Here, indicate whether each material, system or method listed is relevant to your study. If you are not sure if a list item applies to your research, read the appropriate section before selecting a response.

Materials & experimental systems

n/a	Involved in the study	
☒	☐	Antibodies
☒	☐	Eukaryotic cell lines
☒	☐	Palaeontology and archaeology
☐	☒	Animals and other organisms
☒	☐	Human research participants
☒	☐	Clinical data
☒	☐	Dual use research of concern

Methods

n/a	Involved in the study	
☒	☐	ChIP-seq
☒	☐	Flow cytometry
☒	☐	MRI-based neuroimaging

Animals and other organisms

Policy information about studies involving animals; ARRIVE guidelines recommended for reporting animal research

Laboratory animals	No laboratory animals were used in this study

Wild animals	No wild animals were used in this study
Field-collected samples	Stocks of H melpomene rosina and H cydno chioneus were established from individuals collected from the Soberania National Park with permission from the Ministerio del Ambiente, Panama. Butterflies were reared at the Smithsonian Tropical Research Institute insectaries in Gamboa. Larvae were reared on fresh Passiflora shoots/leaves until pupation. Butterflies were reared in 2x2x2m cages, and provided with fresh Psiguria flowers and 10% sugar solution. These cages are maintained on the edge of the butterfly's native habitat and light conditions do not substantially deviate from the forest edge environment.
Ethics oversight	The study did not require ethical approval

Note that full information on the approval of the study protocol must also be provided in the manuscript.
