# Supplementary material for: Visual mate preference evolution during butterfly speciation is linked to neural processing genes
Source: Nat Commun. 2020 Sep 21;11:4763. doi: 10.1038/s41467-020-18609-z (PMC7506007; doi:10.1038/s41467-020-18609-z)
Supplement: Supplementary file 4 — Description of Additional Supplementary Files [file 41467_2020_18609_MOESM4_ESM.pdf]

Description of Additional Supplementary Files

File name: Supplementary Data 1

Description: Sample and sequencing information
