# Supplementary material for: Visual mate preference evolution during butterfly speciation is linked to neural processing genes
Source: Nat Commun. 2020 Sep 21;11:4763. doi: 10.1038/s41467-020-18609-z (PMC7506007; doi:10.1038/s41467-020-18609-z)
Supplement: Supplementary file 2 — Peer Review File [file 41467_2020_18609_MOESM2_ESM.pdf]

Reviewer #1 (Remarks to the Author):

This is a comprehensive dissection of the genes regulating male mating preference behavior in two species of Heliconius butterflies that use visual cues for mate discrimination. The research team combines behavioral measures of male mate preference with differential gene expression, extensive back-crossing to identify genes within the candidate QTL and explore differential expression at different life stages and across eyes and brain tissue. Their findings represent some of the most careful analyses of 'choice' genes, and the results are important contribution to the field.

Summary of their research and findings:
1. Using their refined 3 BC generation approach with individual male preference behavior they had high resolution to identify (1) whether previous putative Heliconius preference candidate gene on the QTL (optix) was modifying male preference behavior, (2) identify other candidate genes involved in regulating differences in male mating preference, (3) determine cis- vs trans-regulation, and (4) compare expression of differentially expressed genes at different developmental time periods.
2. By examining both transcriptomics of different male genotypes (those homozygous or heterozygous for specific parental spp) they could determine which genes on the QTL were associated with specific types of male preference (preference for H. cydno vs H. melpomene).
3. Their study provides compelling evidence against the previous candidate gene putatively linked to male preference (optix which codes for wing coloration).
4. The authors identified 4 candidate genes (within the QTL) that predicted variation in male preference behavior based on his genotype.
5. With this approach, the authors were able to determine whether differential expression was governed by cis- or trans-regulation.
6. The authors present their work as supporting integration or response pathways (associated with synaptic transmission and plasticity) regulating preference behavior (rather than changes in sensory perception).

Importantly, their results suggest that the genes involved in regulating male behavior are not those associated with changing sensory perception, but rather genes involved in male's response to stimuli. Specifically, they found the strongest support for candidate preference genes regulating synaptic transmission, excitability and plasticity. This is important as it differs from some of the previous work that has identified genetic features of preference and sexual traits as having a link to peripheral processes (e.g. cichlid fishes). I think the authors can strengthen their Discussion further by broadening their lens to examine how their results fit into the broader context of mate choice genetic pathways. While their results have begun to solve a mystery in the Heliconious butterflies, but they also start building a larger model for mate choice behavior which extends across taxa and between sexes.
Suggestions:
a. Can the authors present any of their differential expression dataset to compare expression between brains and eyes? The data appears to be graphed as the combination of both tissue, but if the authors have compared gene expression between these two tissues that would be insightful. It would allow the authors to specifically ask if these key gene effects occur in periphery or brain, and it would also allow the authors to use their own transcriptomics to bolster the claim that optix is not expressed in the eye (lines 236-238).

b. Discussion: (lines 227-241) The authors find compelling evidence against the previously suggested hypothesis for optix being differentially expressed in the eyes and regulating male mating preferences by altering male perception of female wing coloration. The authors therefore have data against the 'magic trait' hypothesis (e.g. Servedio et al 2011, TREE), and also against the 'sensory drive' hypothesis that has support in other taxa using vision (e.g. fish; Seehausen etal 2008, Nature), acoustics (crickets: Shaw and Lesnick 2009 PNAS), and chemical (Drosophila: Marcillac et al 2005, Proc Roy Soc B). Given how strong their results are against these hypotheses, I suggest that the authors elaborate further on the fact that their findings standout from these two processes that are

commonly viewed as critical steps toward speciation.

c. Discussion: (lines 242-253) The authors point out that their candidate genes suggest that synaptic transmission pathways (such as the ionotropic glutamate receptor pathway) play a major role in divergent mate preferences across H. cydno and H. melpomene males. However, I believe they could make their discussion stronger by building up this idea as a more general pathway of visual mate choice shared across animals (as well as across the sexes). For instance, in swordtail fishes, neurogenomic examination identified synaptic plasticity candidate genes for female mate preference (Cummings et al 2008 Proc Roy Soc B), closely related species engaged in mate preference exhibit divergent expression of these genes (including ionotropic glutamate receptor, NMDAR; Lynch et al 2012 Genes Brains Behavior), and pharmacological blockade of the NMDAR removed female preference behavior (Ramsey et al 2014 Proc Roy Soc B). These same pathways were also found to be significantly differentially expressed during female mate preference in the closely related guppy (Bloch et al 2018, Nat Ecol Evol). They were also identified in a recent neurogenomic analysis of olfactory mate choice in another swordtail (Declos et al. 2020, J Exp Biol). Given the elegance of the authors' Heliconius work, they are in an excellent position to make a more 'general case' for these pathways having a critical role in mate preference behavior in both sexes and across vertebrates to invertebrates.
Minor Edits:
1. line 61. missing period.
2. line 284. add "than" to read "other than those"
3. Figure 3 legend. I believe the references to standard error bars in the first sentence is reversed. Should read "standard error, ...between parental species (vertical) and the alleles in F1 hybrids (horizontal)." Meanwhile, the sentence noting the dashed lines indicating thresholds, has the horizontal vs vertical correctly assigned.

Reviewer #2 (Remarks to the Author):

This is an amazing dataset and the analyses performed bring original research avenues on the cognitive mechanisms that might be involved in mate choice, as well as evolutionary perspectives on the role of genetic correlation between mate preference and mating cues in driving speciation. Nevertheless the lack of functional validation on the candidate genes identified here prevents to draw solid conclusions on the actual mechanism involved in behavioural changes between these two Heliconius species. In general, I would thus recommend being more careful on the conclusions drawn from the results, by considering alternative hypotheses and by embracing the complexity of species divergence from an ecological point of view (for more details, see specific comments below).

L14-15: "Many animal species remain separate not because they fail to produce viable hybrids, but because they "choose" not to mate".
This sentence must be entirely revised, species do neither fail nor loose anything and even less 'choose' anything. As an evolutionary biologist, I recommend to absolutely avoid any finalistic sentences. Species do not have any aim or success, and the individuals choose mates not the species! This is not only wrongly written but it also conveys misleading ideas on the selective processes involved in the evolution of mate preferences.

L43-44: Changes in the sensitivities towards different wavelength (triggered by variations in opsin genes for instance) have been hypothesized to drive variations in attractions toward different colorations. This could be acknowledged in the introduction, highlighting the alternative hypothesis of the involvement of both downstream but also upstream processes in the evolution of preferences toward visual cues. Moreover, species recognition can also involve a combination of visual, odorant and behavioural cues, and this should be better explained.

L46: 'selection for mimicry' is a again a finalistic sentence and should be rephrased.

L50: Preferences are usually expected to be stronger in females, because of their greater investment in reproduction. Although the spermatophore transferred by male butterflies might incur an important metabolic cost, it is still unclear whether or not males are choosier than females in Heliconius, notably because female preferences are infrequently tested. For instance, note that in Chouteau et al PNAS 2017, female and male choice wer tested and females were found choosier. This should be clarified in the manuscript.

L51-52 and hereafter (L56, L72): add 'H.'before 'melpomene' and 'cydno', this is important to respect conventions regarding species names in scientific articles.

L57-58: The expression of the gene optix is involved in the variations of red pattern, but is there any evidence of its role in the switch between red and white patterns ? You may cite Martin et al. EvoDevo 2014 to argue about the cis-regulation of optix in H. cydno.

L59: This is misleading: when an ecological trait is used as a mating cue, it can be a 'magic' trait favouring speciation. Then the linkage desquilibrium between the locus controlling the 'magic' trait and the locus controlling the preference toward this 'magic' trait further promote the divergent evolution of the trait and may ultimately promote speciation. There is a mix here, this should be properly explained.
The same lack of precision is also in the discussion (L 218-219) and should be corrected.

L72-73: The behaviour of males towards the females depends on the wing colour pattern which is strikingly different between H. melpomene and H. cydno, but given that the experiment was carried out with live females, other cues might be involved (flying behaviour, pheromones). So that the preference variation detected here could also involve other pathway than the visual one? How much this would affect the result reported here ? For instance, you focused on the RNA expression in the brain and in the eye and not in other tissue like the antennae. Furthermore, the cydno/melpomene QTL allele was inferred from the wing colour pattern, but if other cues unrelated to coloration are involved, their causal genes might be located elsewhere in the genome. I would recommend checking the results in light of this alternative (not mutually exclusive) hypothesis.

L104-105: How much the differential expression detected in chromosome 18 differs from the differential expression observed on the other chromosomes? It would be more convincing to provide a statistical test investigating whether differential expression is higher in this chromosomes as compared to the others ?

L196-197: The grik2 alleles of cydno and melpomene thus do not exhibit any changes that could be predicted to trigger a functional difference in the resulting protein ?

L208-210: From fig.4, we can see than Grik2 is located in a region of low fd (although the fd value in this region seems higher than the fd value around optix) but it is really not obvious for the regucalcin genes. Could you provide a statistical test to estimate the confidence of this effect?

L214: "Behavioural isolation is frequently implicated in the formation of new species, and involves the correlated evolution of both mating cues and mating preference" You may provide references, I am not sure that speciation events driven by behavioural isolation, without any ecological adaptation associated with the mating cue, are that common. This should be clarified.

L223-224: How could you interpret the lack of overlap in the candidates identified with gene expression analysis and functional analysis?

L269-276: The lack of evidence of functional or regulatory links among the different candidate genes

prevents any robust conclusions on their putative role in variations of mate preferences. Without solid functional validation on the role of these candidates on preference variations, it seems risky to draw robust conclusions on the molecular and/cognitive mechanism involved in these behavioural variations.

L302-304: The selective constraint on the evolution of visual perception putatively imposed by the adaptation to the 'wider' environment is not demonstrated. Speciation is likely to be driven by multiple changes in ecological niches (mimicry ring, micro-habitats, mate preference) that are likely to evolve in concert. I would recommend being careful not draw too simplistic hypotheses without evidences on ecological divergence involved.

I had some troubles interpreting the figure 1, if Left ternary axis shows proportion of trials where courtship was initiated towards H. cydno female only, bottom axis towards H. melpomene female only, and right axis towards both female species, what is the behavior reported in circles located outside from the axis ? Each circle shows the behaviour of one individual during the 5 trials? What are the units of the axis? How is it standardized?
The reader needs a bit more detail in the caption to really capture the results reported in figure 1. Is the general trend toward a more marked preference of cydno males toward cydno females than the preference of melpomene toward melpomene females? (as shown by the greater number of blue circles on the left ternary axis than the number of orange circles on the right ternary axis ?) If so, this should also be better explained in the text of the manuscript.

Reviewer #3 (Remarks to the Author):

QTL mapping of traits like behavior is a much-needed area of research in speciation. This is an exciting system for this type of genetic dissection given the well-described genetic basis for wing pattern differences and detailed mate choice studies conducted by the authors and colleagues. The analyses in the paper are thorough and the presentation of the data and clarity of writing is excellent.

I think the backcross experiment is a key reason why I find this manuscript compelling, but I did not find it well-integrated into the manuscript. Without the backcross experiment, I find the focus on differentially expressed genes (DE genes) within the QTL interval on Chr 18 dissatisfying. It is just as likely that the QTL interval contains a trans-acting regulatory element that would be missed by focusing solely on candidate genes within that interval. The backcross experiment essentially tests whether a mel allele at the Chr 18 QTL impacts expression anywhere in a mostly cyd genome, and if I am interpreting this correctly, then the lack of DE genes outside of the QTL interval suggests there are no trans effects of the Chr 18 QTL? If that is correct, then I think this point should be made more explicitly in the manuscript.

In general, the compactness of the manuscript means a few details hard to follow. It wasn't clear to me if the two candidate genes were differentially expressed across all developmental stages (it is hard to distinguish individual genes in Figure 2). How does this relate to the BC where only pupal stages were sequenced? The results should clarify which BC samples are being compared (which is not presented until the methods, Lines 456-459). After three generations of backcrossing, what was the average genome composition (outside of Chr 18, Lines 454-455)? I indicated other details that might be helpful in my comments below.

While the ASE analyses provide some support for the cis regulatory change, they are not particularly conclusive. How low were the number of reads that mapped to diagnostic SNPs and was that read mapping sufficient for the analyses (this might require a power analysis?). ASEReadCounter filters out duplicate reads by default, and it looks like the default settings were used for this analysis (Line 471). This is a strange default setting given that RNAseq datasets typically have many reads that are

identical due to real expression differences. Could this have impacted the number of reads for the ASE mapping? If there are insufficient reads in the total RNAseq dataset, why not use targeted sequencing of these genes to estimate ASE? Considering other regulatory categories may also be helpful (e.g., cis + trans, cis x trans, compensatory) given the differences in cis vs trans changes between adult and pupal stages. I recognize these cutoffs are fuzzy and somewhat arbitrary, but if I am looking at Figure 3 correctly, it looks like Grik2 may be cis+trans given that the mel allele has higher expression in the parents than in than in the hybrids. At the very least, I think it is worth noting that with low sequencing depth, whether Grik2 is cis/trans or both at different stages is not exactly clear.

Minor Comments:

Line 36-37: To be fair, the male calling song and female preference in crickets has now been mapped in a QTL study with a very promising candidate gene, a nucleotide-gated ion channel gene (Xu and Shaw, 2019, Genetics). This study should be cited in this paragraph.

Line 58: It would be nice to integrate the sexual selection literature here – signals and preferences are predicted to be linked.

Line 86: It might be worth mentioning here that collectively these results suggest the mel allele is dominant, since this is referred to later in the manuscript (Line 124).

Line 93: Supplemental Table 1 A reports DE results for Chr 1, so why is this cited here for QTL analysis?

Line 100: Maybe change imago to adult to avoid unnecessary entomology jargon?

Line 106: How many genes were DE across the rest of the genome, and are there more/less DE genes on Chr 18 than expected compared to other autosomes?

Line 114: It is a bit weird to refer to mapping bias as from divergent coding sequences.. any SNPs can cause mapping bias?

Lines 115-116 and Line 43 in Supplement: Could the manuscript include the number of reads mapped to each reference for each sample?

Line 128: There were no genes on Chr 1 that were DE between F1 hybrids and cydno?

Line 140: It could be worth clarifying here that the expectation is that a DE gene within the QTL interval would be regulated via cis change if that DE gene is associated with the QTL.

Line 163: Was DE analysis for the BC done only on pupal stages (i.e. not adults)?

Line 348: Perhaps clearer to keep referring to this introgression line as a backcross?

Line 392: There are differences in the cell composition/structure of the brains between species? Perhaps mention this in the discussion?

Figure 2: There is fuchsia vertical line indicating a gene that was excluded because it did not show differential expression or showed the reversal fold change when mapping to the cydno genome. Given the proximity of this gene to the DE genes identified as candidates, could this be clarified? Is this because there was less power to identify candidate genes with the cydno genome? Or is it because there is divergence or structural reasons why reads are mapping differently in that region?

Authors' remark

We have conducted a new, independent RNA-seq experiment on a further 5 *H. melpomene, 5 H. cydno,* and 6 F1 hybrids adult males. We repeated the differential expression analyses reported in the previous version of the manuscript (both analysing the old and new datasets separately, and together): the candidate genes *Grik2* and *regucalcin2* were consistently detected as differentially expressed between *H. melpomene* and *H. cydno*, and between F1 hybrids and *H. cydno,* across these analyses. The F1 hybrids also allowed a more powerful allele-specific expression analysis. We have added sections relating to these new data/analyses to the Results at Line 155-161 and in the Methods at Line 438-444.

Reviewer #1 (Remarks to the Author)

This is a comprehensive dissection of the genes regulating male mating preference behavior in two species of Heliconius butterflies that use visual cues for mate discrimination. The research team combines behavioral measures of male mate preference with differential gene expression, extensive back-crossing to identify genes within the candidate QTL and explore differential expression at different life stages and across eyes and brain tissue. Their findings represent some of the most careful analyses of 'choice' genes, and the results are an important contribution to the field.

Summary of their research and findings:
1. Using their refined 3 BC generation approach with individual male preference behavior they had high resolution to identify (1) whether previous putative Heliconius preference candidate gene on the QTL (optix) was modifying male preference behavior, (2) identify other candidate genes involved in regulating differences in male mating preference, (3) determine cis- vs trans-regulation, and (4) compare expression of differentially expressed genes at different developmental time periods.
2. By examining both transcriptomics of different male genotypes (those homozygous or heterozygous for specific parental spp) they could determine which genes on the QTL were associated with specific types of male preference (preference for H. cydno vs H. melpomene).
3. Their study provides compelling evidence against the previous candidate gene putatively linked to male preference (optix which codes for wing coloration).
4. The authors identified 4 candidate genes (within the QTL) that predicted variation in male preference behavior based on his genotype.
5. With this approach, the authors were able to determine whether differential expression was governed by cis- or trans-regulation.
6. The authors present their work as supporting integration or response pathways (associated with synaptic transmission and plasticity) regulating preference behavior (rather than changes in sensory perception)

Importantly, their results suggest that the genes involved in regulating male behavior are not those associated with changing sensory perception, but rather genes involved in male's response to stimuli. Specifically, they found the strongest support for candidate preference genes regulating synaptic transmission, excitability and plasticity. This is important as it differs from some of the previous work that has identified genetic features of preference and sexual traits as having a link to peripheral processes (e.g. cichlid fishes). I think the authors can strengthen their Discussion further by broadening their lens to examine how their results fit into the broader context of mate choice genetic pathways. While their results have begun to solve a mystery in the Heliconius butterflies, but they also start building a larger model for mate choice behavior which extends across taxa and between sexes.

We thank the reviewer for the positive comments and useful suggestions.

Suggestions:

a. Can the authors present any of their differential expression dataset to compare expression between brains and eyes? The data appears to be graphed as the combination of both tissue, but if the authors have compared gene expression between these two tissues that would be insightful. It would allow the authors to specifically ask if these key gene effects occur in periphery or brain, and it would also allow the authors to use their own transcriptomics to bolster the claim that optix is not expressed in the eye (lines 236-238).

We now explain more clearly in the manuscript that eyes (ommatidia and retinal membrane) and brain were not detached, but dissected (and RNA-sequenced) as a single combined tissue at L418:

"Eye (ommatidia and retinal membrane) and brain tissue (central brain and optic lobes) were dissected out of the head capsule (as a single combined tissue)"

We agree with the reviewer that separating eye and brain tissue might help distinguish between expression of genes in the sensory periphery or in higher-processing centres, and thought about this when designing the RNA-seq experiments. Ultimately, however, we decided not to separate eye and brain tissue: combining eye and brain allowed us to optimise the number of biological replicates (across developmental stages), increasing power to detect differences, as well the reliability of our results. Furthermore, the "periphery" tissue would probably have to include the lamina, the outer part of the optic lobe, because the retinal membrane is strongly attached to it, making any inferences about expression in the sensory 'periphery' vs 'brain' inconclusive.

With regard to the claim that *optix* is not expressed in the eyes we cite (L279) a study by Martin et al. (*Evo Devo* 2014) which did not detect the *optix* product in the retinas of different *Heliconius* species, including *H. melpomene* and *H. cydno*.

b. Discussion: (lines 227-241) The authors find compelling evidence against the previously suggested hypothesis for optix being differentially expressed in the eyes and regulating male mating preferences by altering male perception of female wing coloration. The authors therefore have data against the 'magic trait' hypothesis (e.g. Servedio et al 2011, TREE), and also against the 'sensory drive' hypothesis that has support in other taxa using vision (e.g. fish; Seehausen et al 2008, Nature), acoustics (crickets: Shaw and Lesnick 2009 PNAS), and chemical (Drosophila: Marcillac et al 2005, Proc Roy Soc B). Given how strong their results are against these hypotheses, I suggest that the authors elaborate further on the fact that their findings standout from these two processes that are commonly viewed as critical steps toward speciation.

Although we argue that "it seems unlikely … that changes in cue and preference are pleiotropic effects of the same allele" (L336-337), we disagree with the reviewer that this represents 'data against the magic trait hypothesis'. Wing patterns are under both divergent ecological selection through aposematism and are cues for mating, thereby coupling two components of reproductive isolation. Nevertheless, we do think this is an important point that should be clarified, and as such we have now added the following to the introduction (L49-51):

"The closely related species *Heliconius melpomene* and *H. cydno* differ in warning patterns, which are both under disruptive selection due to mimicry[15] and are important mating cues[16]. As a result, these divergent patterns couple ecological and behavioural components of reproductive isolation, which (as predicted by so-called "magic trait" models[17]) is expected to facilitate speciation in the face of gene flow"

We think the reviewer may be referring to an "automatic magic trait" (as opposed to a 'classic magic trait', *sensu* Servedio *et al.* 2011), which – in this specific case – *would* require colour pattern alleles to have pleiotropic effects on warning pattern *and* mating preferences. Citing our previous results (Merrill *et al* 2012; Jiggins *et al* 2001), Servedio *et al.* (2011) argue that colour patterns in *Heliconius* is a well supported example of a (classic) magic trait ("In only one case have both criteria been met by manipulative experiments" p391). We agree with this interpretation, and believe it has likely been important, if not critical, for speciation in these butterflies (by helping to maintain LD between ecological and behavioural components of reproductive isolation).

Our results suggest the underlying mechanism is a change in visual processing rather than detection at the periphery, which might allow preferences to evolve without changing perception of the wider environment; however, we do not think this in itself is evidence against sensory drive (it is still possible that change in the visual processing centres may be driven by adaptation to the sensory environment).

Nevertheless, we agree that sensory drive is unlikely to explain divergent preferences in *Heliconius* (in the sense that they might result from adaptation to meet the *sensory* needs of the local environment). Both species, for example, feed on red flowers, and although it remains to be tested how these butterflies use colour to find food, and how this might vary between deep forest (*H. cydno*) and forest edge (*H. melpomene*) environments, we would expect some degree of constancy in colour perception (as has been shown for other lepidoptera: Kinoshita & Arikawa 2000 *J. Exp. Bio*l. Balkenius & Kelber 2004 *J. Exp. Biol.).* Perhaps more importantly, very strong selection acts on warning patterns in these species, and it seems unlikely that sexual selection imposed by male preferences will have much influence on their evolution (especially given that the operational sex ratio is highly skewed towards males). As we have discussed elsewhere (Merrill et al 2019 *PLoS Biol*), it seems much more likely that behavioural isolation results from divergence in female (and male) wing patterns due to mimicry, which subsequently imposes divergent sexual selection on male preferences to improve their ability to find receptive females.

Overall, we feel that further discussion of sensory drive models of speciation, and how they might relate to *Heliconius*, would likely be a distraction, and beyond the scope of this manuscript.

c. Discussion: (lines 242-253) The authors point out that their candidate genes suggest that synaptic transmission pathways (such as the ionotropic glutamate receptor pathway) play a major role in divergent mate preferences across H. cydno and H. melpomene males. However, I believe they could make their discussion stronger by building up this idea as a more general pathway of visual mate choice shared across animals (as well as across the sexes). For instance, in swordtail fishes, neurogenomic examination identified synaptic plasticity candidate genes for female mate preference (Cummings et al 2008 Proc Roy Soc B), closely related species engaged in mate preference exhibit divergent expression of these genes (including ionotropic glutamate receptor, NMDAR; Lynch et al 2012 Genes Brains Behavior), and pharmacological blockade of the NMDAR removed female preference behavior (Ramsey et al 2014 Proc Roy Soc B). These same pathways were also found to be significantly differentially expressed during female mate preference in the closely related guppy (Bloch et al 2018, Nat Ecol Evol). They were also identified in a recent neurogenomic analysis of olfactory mate choice in another swordtail (Declos et al. 2020, J Exp Biol). Given the elegance of the authors' Heliconius work, they are in an excellent position to make a more 'general case' for these pathways having a critical role in mate preference behavior in both sexes and across vertebrates to invertebrates.

We thank the reviewer for these suggestions and pointing us to relevant literature. We agree that their inclusion would make the discussion stronger and have now expanded on the associations between ionotropic receptors and mate preferences (L290-294), integrating some of the suggested references:

"differential expression of ionotropic receptors is also associated with variation in female preference behaviours in fish[50,51,52], raising the possibility that ion channels might provide a likely route to modulate mate preferences across taxa more broadly"

Minor Edits:
1. line 61. missing period.

Added.

2. line 284. add "than" to read "other than those"

Added.

3. Figure 3 legend. I believe the references to standard error bars in the first sentence are reversed. Should read "standard error, ...between parental species (vertical) and the alleles in F1 hybrids (horizontal)." Meanwhile, the sentence noting the dashed lines indicating thresholds, has the horizontal vs vertical correctly assigned.

We thank the reviewer for pointing this out, we have now corrected this sentence.

Reviewer #2 (Remarks to the Author):

This is an amazing dataset and the analyses performed bring original research avenues on the cognitive mechanisms that might be involved in mate choice, as well as evolutionary perspectives on the role of genetic correlation between mate preference and mating cues in driving speciation. Nevertheless, the lack of functional validation on the candidate genes identified here prevents to draw solid conclusions on the actual mechanism involved in behavioural changes between these two Heliconius species. In general, I would thus recommend being more careful on the conclusions drawn from the results, by considering alternative hypotheses and by embracing the complexity of species divergence from an ecological point of view (for more details, see specific comments below).

We thank the reviewer for the positive comments regarding our data and analyses and for useful suggestions.

L14-15: "Many animal species remain separate not because they fail to produce viable hybrids, but because they "choose" not to mate". This sentence must be entirely revised, species do neither fail nor loose anything and even less 'choose' anything. As an evolutionary biologist, I recommend to absolutely avoid any finalistic sentences. Species do not have any aim or success, and the individuals choose mates not the species! This is not only wrongly written but it also conveys misleading ideas on the selective processes involved in the evolution of mate preferences.

We did not intend to confer species a "choosing entity" status, but we acknowledge the phrasing might be confusing, and we have changed L15 which now reads:

"but because their individuals "choose" not to mate".

L43-44: Changes in the sensitivities towards different wavelengths (triggered by variations in opsin genes for instance) have been hypothesized to drive variations in attractions toward different colorations. This could be acknowledged in the introduction, highlighting the

alternative hypothesis of the involvement of both downstream but also upstream processes in the evolution of preferences toward visual cues.

We have elaborated on L44 as follows:

"[..] both suggesting a role for sensory perception mediated by changes in the peripheral visual system (e.g. opsins)".

Moreover, species recognition can also involve a combination of visual, odorant and behavioural cues, and this should be better explained.

We agree with the reviewer that multiple traits may be important in the overall 'decision' to mate. However, previous work (Jiggins et al. 2001 Nature, Jiggins et al. 2006 *J. Evol. Biol.*, Merrill et al. 2012 *Proc. R. Soc. B Biol. Sci.*) demonstrates that colour pattern preference plays a dominant role in this species pair. Our aim here is to identify genes underlying preference for this *specific* component of mate choice. (We expand on this point further below.)

L46: 'selection for mimicry' is again a finalistic sentence and should be rephrased.

We have changed this so it now reads "disruptive selection due to mimicry"

L50: Preferences are usually expected to be stronger in females, because of their greater investment in reproduction. Although the spermatophore transferred by male butterflies might incur an important metabolic cost, it is still unclear whether or not males are choosier than females in Heliconius, notably because female preferences are infrequently tested. For instance, note that in Chouteau et al PNAS 2017, female and male choice were tested and females were found choosier. This should be clarified in the manuscript.

Female choice exists (and is likely important) in *Heliconius* and we agree that more clearly acknowledging this would improve the discussion. We have now added a paragraph (with a focus on the possible genetic mechanisms shared by males and females), at Lines 309-321, that reads:

"Neither *regucalcin2* or *Grik2* show male-biased gene expression, which might be expected of candidate genes for a behaviour that is evident only in males. It is possible that the lack of sex-biased expression indicates that visual cues are similarly important in female mating preference in this species pair. Indeed, Chouteau *et al*[59] report that female preferences contribute to *disassortative* mating between colour pattern morphs of *H. numata,* and it is possible that in *H. cydno* and *H. melpomene* females share the same genetic basis for colour pattern-based discrimination as males. Alternatively, if visual preference behaviour is restricted to males, changes in gene expression may be integrated differently in the female and male nervous systems. The role of female preference in *Heliconus* mate choice remains poorly understood. Although emerging data suggests that female choice does contribute to reproductive isolation between *melpomene-cydno* clade taxa[60,61], it remains to be tested if there is a strong visual component to this preference similar to that observed in males."

Although sexual selection theory predicts preferences to be more pronounced in females, strong male preferences have nevertheless been repeatedly demonstrated in *H. cydno* and *H. melpomene* (as well as more broadly across *Heliconius*). In addition, in these species, male preferences act before any potential female choice could take place such that strong male discrimination against heterospecific females will have a disproportionate contribution to overall reproductive isolation (see Jiggins *Biosciences* 2006 and Merrill *PLoS Biol.* 2019 for a specific discussion on how the

order in which barriers act influence reproductive isolation in *H. cydno* and *H. melpomene*).

L51-52 and hereafter (L56, L72): add 'H.'before 'melpomene' and 'cydno', this is important to respect conventions regarding species names in scientific articles.

We have corrected this throughout the manuscript.

L57-58: The expression of the gene optix is involved in the variations of red pattern, but is there any evidence of its role in the switch between red and white patterns ? You may cite Martin et al. EvoDevo 2014 to argue about the cis-regulation of optix in H. cydno.

We have modified Line 62, so that it now reads: "presence and absence of red colour pattern elements in *Heliconius*", based on the following reasoning: While *optix* determines the presence of the red elements in *Heliconius,* and the lack of *optix* expression is correlated with absence of red elements in *H. cydno*, other features of the forewing pattern (e.g. shape) vary across *Heliconius* (for example other loci influence where the boundary between red and white will be in the forewing band), so we think that saying that *optix* determines the switch between the red and white pattern is not strictly correct, because it implies that *optix* governs all the different features of the forewing pattern.

L59: This is misleading: when an ecological trait is used as a mating cue, it can be a 'magic' trait favouring speciation. Then the linkage disequilibrium between the locus controlling the 'magic' trait and the locus controlling the preference toward this 'magic' trait further promotes the divergent evolution of the trait and may ultimately promote speciation. There is a mix here, this should be properly explained. The same lack of precision is also in the discussion (L 218-219) and should be corrected.

We agree that colour pattern is a 'magic trait' and that this will facilitate speciation, and now explicitly state this in the introduction (L48-51):

"The closely related species *Heliconius melpomene* and *H. cydno* differ in warning patterns, which are both under disruptive selection due to mimicry[15] and are important mating cues[16]. As a result, these divergent patterns couple ecological and behavioural components of reproductive isolation, which (as predicted by so-called "magic trait" models [17]) is expected to facilitate speciation in the face of gene flow".

However, we are not sure why the reviewer considers these particular lines misleading. The existence of "magic traits" will not inevitably lead to speciation, which will further be influenced by the genetic architecture of the traits involved. In particular: "Genetic linkage will facilitate speciation by impeding the breakdown of genetic associations between ecological and mating traits". We don't see the two mechanisms as mutually exclusive.

L72-73: The behaviour of males towards the females depends on the wing colour pattern which is strikingly different between H. melpomene and H. cydno, but given that the experiment was carried out with live females, other cues might be involved (flying behaviour, pheromones). So that the preference variation detected here could also involve other pathway than the visual one? How much this would affect the result reported here ? For instance, you focused on the RNA expression in the brain and in the eye and not in other tissue like the antennae.

Extensive previous work (Jiggins et al. 2001 Nature, Jiggins et al. 2006 *J. Evol. Biol.*, Merrill et al. 2012 *Proc. R. Soc. B Biol. Sci.*), demonstrates that visual cues play a dominant role for male mate preference in *H. melpomene and H. cydno* (and between

other *Heliconius* taxa). Our previous results (Merrill et al. 2019 PLoS Biol.) reveal that the QTL on chromosome 18 is both very well supported and is of large effect, and here we demonstrate that this QTL influences courtship initiation. As such, and given the importance of visual cues for male mate preference in these species - we considered this QTL region an excellent place to search for candidates underlying *visual* preference evolution - hence our focus on this region. In addition, as stated at L95-98, our "[..] previous evidence [suggests] that male hybrids bearing *H. melpomene* alleles at *optix* prefer to court the artificial models of *H. melpomene* females over those of *H. cydno* (Merrill et al 2012 *Proc. R. Soc. B Biol. Sci*)", further implicating this region in *visual* mate preference.

We also think it is unlikely that other cues (e.g. pheromones) influence the results for courtship initiation analysis presented in this manuscript (Figure 1). In particular, by 'explicitly considering whether males *initiated* courtship' (i.e. males either courted a female in the trial or did not), we are reasonably confident that these analyses capture initial (*i.e.* longer range) attraction to females, which is likely a response to visual cues. It is perhaps worth noting that *Heliconius* lack specialized olfactory structures to support long range detection of chemical signals so are only likely to use these in close proximity.

A recent study (van Schooten et al. 2020 *PNAS*) also found no candidate chemosensory genes for reproductive isolation at this QTL (or any other QTL), further implicating the visual component of QTL on chr 18. We have added this at L101-102:

"A recent study also reports no candidate chemosensory genes for reproductive isolation at this QTL region on chromosome 18 (or any other QTL)."

That said, we do broadly agree with the reviewer that multiple cues, acting across different sensory modalities, may affect *Heliconius* courtship. It is conceivable that the QTL on chromosome 18 (or other QTL including those on chromosomes 1 and especially 17), may additionally influence the detection/processing of other cues that influence mating preferences, but we think this is beyond the scope of the current manuscript. We point out that, except for the periphery of the odor pathway, the type of tissue sampled in this study should eventually capture all possible odor-processing related changes (e.g. in the antennal lobe, lateral horn, etc.).

Furthermore, the cydno/melpomene QTL allele was inferred from the wing colour pattern, but if other cues unrelated to coloration are involved, their causal genes might be located elsewhere in the genome. I would recommend checking the results in light of this alternative (not mutually exclusive) hypothesis.

We are not sure what the reviewer means here. Perhaps the reviewer is referring to the tracking of *optix* alleles in the introgression line? It is true that in the backcrosses we tracked the alleles on chr18 based on presence/absence of the red band, but as a Mendelian trait it can be used to perfectly infer genotype at the *optix* locus. Arguably, this is not the same as tracking the preference QTL - however, *optix* is within the QTL confidence region, and is very tightly linked (1.2cM) to markers at the QTL peak. As a result, any recombination between these two loci would be very rare (we would expect only ~0.04 recombination events across the three generations of backcrossing), but in any case, the allele status at the QTL was inferred from genetic data in the third-generation backcross hybrid samples, as we point out at L413-415:

"Due to the tight linkage we expected little recombination between *optix* and QTL peak even after three generations of introgression, allowing us to infer genotype at the preference-*optix* locus (which we confirmed with genetic data, see below)."

These analyses also reveal that the other known QTL (on chromosome 1 and 17) were not introgressed. Even if backcrosses differ for other genetic variants affecting behavioural preference (likely of small effect), this would probably not affect the action of causal genes on 18, which we set to investigate with these backcrosses (and the manuscript in general as discussed above).

Alternatively, perhaps the reviewer is referring to the QTLs themselves. These were identified using a linkage map generated from genome-wide RADseq data as described in our previous paper (Merrill et al 2019 PLoS Biology), and these same markers (at the QTL peaks) are used for the behavioural analyses presented in Figure 1 (*i.e.* we infer *cyd*/*cyd* or *cyd*/*melp* genotype based on genetic markers at the QTL peak). As such the identification of the QTLs influencing male preference behaviour, and our subsequent behavioural analyses here, were independent of the location of the colour pattern genes.

We apologise if we have misunderstood the reviewer's point, but hope our response addresses their concerns.

L104-105: How much the differential expression detected in chromosome 18 differs from the differential expression observed on the other chromosomes? It would be more convincing to provide a statistical test investigating whether differential expression is higher in this chromosomes as compared to the others?

Our intention here was not to suggest that gene expression is higher in this region compared to other parts of the genome, but simply that there were a number of differentially expressed genes within the focal QTL which explains a large proportion of variation in visual mate preference. To avoid this confusion, we have changed these sentences so that they now read:

"Across the QTL region on chromosome 18 (which spans 2.75 Mb and contains 200 genes), we identified 27 genes that show differential expression between *H. melpomene* and *H. cydno*, in at least one of the three developmental stages."

Chromosome 18 is not enriched for differentially expressed genes (Fisher's Exact test: $P > 0.05$, for all three stages), though we don't think this is relevant for our results (and would expect a large number of differentially expressed genes across the genome).

L196-197: The grik2 alleles of cydno and melpomene thus do not exhibit any changes that could be predicted to trigger a functional difference in the resulting protein?

Yes - according to the PROVEAN analysis, only the four genes listed at L239-243 are predicted to have changes that have functional effects on proteins.

L208-210: From fig.4, we can see that Grik2 is located in a region of low fd (although the fd value in this region seems higher than the fd value around optix) but it is really not obvious for the regucalcin genes. Could you provide a statistical test to estimate the confidence of this effect?

Based on these data, we agree with the reviewer that *Grik2* might be considered a better candidate than the other genes. However, regulatory elements influencing gene expression, and that of *regucalcin2* in particular, may not be directly adjacent to the gene itself (Klenjan & van Heyningen 2005 Am. J. Hum. Genet, Kwon et al. 2019 Development). In addition, it may be worth noting that, although we expect fd to be reduced, there is no clear expectation on how strong the effect on fd should be for

preference loci (which in contrast to the colour pattern loci may only be maintained by indirect selection)*.*

We know of no statistical test that would estimate the confidence of these effects, although we now indicated the 5% and 25% quantiles of the genome-wide fd distribution in Figure 4. In general, these analyses only reveal that the loci of interest are in regions of reduced admixture, and should probably be taken as observations *consistent* with our hypotheses, rather than a direct test.

L214: "Behavioural isolation is frequently implicated in the formation of new species, and involves the correlated evolution of both mating cues and mating preference" You may provide references, I am not sure that speciation events driven by behavioural isolation, without any ecological adaptation associated with the mating cue, are that common. This should be clarified.

We agree with the reviewer that it is unlikely that 'speciation events [are] driven by behavioural isolation' alone. However, we maintain that this is not what we suggest: "Behavioural isolation is *'frequently implicated'* in speciation" (including models of ecological speciation). We have now included a citation to two monographs Coyne and Orr 2004 and Nosil 2012.

L223-224: How could you interpret the lack of overlap in the candidates identified with gene expression analysis and functional analysis?

'Functional analysis' carried out with PROVEAN identifies changes in genes' coding sequences. These changes, and changes in gene expression, are two alternative mechanisms that might underlie phenotypic change, and although they are not mutually exclusive, we do not necessarily expect overlap.

We suppose that one possible interpretation might be that genes that show expression differences are pleiotropic, and so protein-coding changes are more likely to be detrimental for these genes (we do argue these are likely to be pleiotropic, but this is based on predicted gene function). However, we don't think this is a robust line of inference: following the same reasoning, all genes not showing protein-coding changes predicted to alter protein function could be inferred to be pleiotropic, but we think there is no evidence to support this claim.

L269-276: The lack of evidence of functional or regulatory links among the different candidate genes prevents any robust conclusions on their putative role in variations of mate preferences. Without solid functional validation on the role of these candidates on preference variations, it seems risky to draw robust conclusions on the molecular and/cognitive mechanism involved in these behavioural variations.

We agree that caution should be exercised, and our intended point is that we cannot exclude these genes based solely on associated function, as very little is known on gene functions associated with behavioural evolution.

With regards to the broader point that 'it seems risky to draw robust conclusions on the molecular and/cognitive mechanism involved in these behavioural variations', neither QTL modulating male attraction behaviours on Chr 1 and 18 contain opsins, and none of the candidates have an obvious relation to light detection/the first steps of the phototransduction cascade (Montell 2012, Trends Neurosci). We agree that we cannot completely rule out that this divergent behaviour relies on divergent sensory reception, and although we maintain that our results do strongly suggest genetic changes affecting processing rather than light detection – we think we have used cautious language in our conclusion:

“candidate genes identified seem *more likely* to alter visual processing … , *consistent with* …” (L358-360)

L302-304: The selective constraint on the evolution of visual perception putatively imposed by the adaptation to the ‘wider’ environment is not demonstrated. Speciation is likely to be driven by multiple changes in ecological niches (mimicry ring, micro-habitats, mate preference) that are likely to evolve in concert. I would recommend being careful not to draw too simplistic hypotheses without evidence on ecological divergence involved.

There is evidence for ecological divergence between *H. cydno* and *H. melpomene*: They are isolated across a gradient of open to closed forest (Estrada & Jiggins 2002 Eco. Ento.) and of decreasing light intensity (Seymoure et al. 2015, J. Res. Lepid.).

We agree that selection imposed by the broader environment (i.e. other than that involved in mate choice) on visual perception in these butterflies has to be demonstrated. Nevertheless, we think it is fair to assume that visual perception of, for example, colour or brightness, is likely to be important for fundamental tasks like finding food or host plants, or more generally to navigate the environment. Therefore, we posit that visual perception of mating cues is likely to be dependent on how the visual system is shaped by the wider environment.

I had some troubles interpreting the figure 1, if Left ternary axis shows proportion of trials where courtship was initiated towards H. cydno female only, bottom axis towards H. melpomene female only, and right axis towards both female species, what is the behavior reported in circles located outside from the axis? Each circle shows the behaviour of one individual during the 5 trials? What are the units of the axis? How is it standardized?
The reader needs a bit more detail in the caption to really capture the results reported in figure 1.

We have elaborated the Figure 1 legend, which now reads as follows:

Figure 1. Genotype at the preference QTL on chromosome 18 influences courtship initiation. [...]. Each point represents an individual and the location of the point in the triangle is a way of representing these three proportions at once. Lines project the three predicted proportions to corresponding values on the three axes and 95% credibility intervals (CrIs) for these proportions are shown as hexagons. [...] Point size is scaled to the number of trials in which the male showed a response and a ‘jitter’ function has been applied (leading to some points being jittered to outside the triangle).

Is the general trend toward a more marked preference of cydno males toward cydno females than the preference of melpomene toward melpomene females? (as shown by the greater number of blue circles on the left ternary axis than the number of orange circles on the right ternary axis?) If so, this should also be better explained in the text of the manuscript.

We have changed at L84 as follows:

“Consistent with our previous analyses[20], we found that males of both species, in particular *melpomene* males, show a strong preference for females of their own phenotype. F1 and backcross-to-*melpomene* prefer to court *melpomene* females [...] (Figure 1).”

Reviewer #3 (Remarks to the Author):

QTL mapping of traits like behavior is a much-needed area of research in speciation. This is an exciting system for this type of genetic dissection given the well-described genetic basis for wing pattern differences and detailed mate choice studies conducted by the authors and colleagues. The analyses in the paper are thorough and the presentation of the data and clarity of writing is excellent. I think the backcross experiment is a key reason why I find this manuscript compelling, but I did not find it well-integrated into the manuscript. Without the backcross experiment, I find the focus on differentially expressed genes (DE genes) within the QTL interval on Chr 18 dissatisfying.

We appreciate the reviewer's positive comments about the manuscript. We agree with the reviewer that the backcrosses constitute an important part of the dataset, which we think complement the pure species data. In the newer version of the manuscript we have tried to better integrate these results.

It is just as likely that the QTL interval contains a trans-acting regulatory element that would be missed by focusing solely on candidate genes within that interval. The backcross experiment essentially tests whether a mel allele at the Chr 18 QTL impacts expression anywhere in a mostly cyd genome, and if I am interpreting this correctly, then the lack of DE genes outside of the QTL interval suggests there are no trans effects of the Chr 18 QTL? If that is correct, then I think this point should be made more explicitly in the manuscript.

Outside the QTL interval, as we now state more explicitly (L196-199), there are 10 genes that were differentially expressed between both species and backcross hybrids (at either stage), of these:

"seven were located within the introgressed region (0 - ~6.3Mb, Supplementary table S3), and it seems most likely that these are regulated by introgressed *H. melpomene cis*-acting elements."

It is of course possible that the causal gene/s within the QTL could act on other loci in *trans* (both on chromosome 18 and elsewhere in the genome), and the reviewer is correct that our backcrosses allow us to consider this. In this newer version of the manuscript we discuss this in more detail (L202):

"It is possible that causative loci (e.g. expressed RNA or protein factors) within the QTL could act on other genes in *trans* (both on chromosome 18 and elsewhere in the genome), and identifying these could provide insight into the mode of action of causative genetic elements. Three genes differentially expressed both in species and backcross hybrid comparisons, and located outside of introgressed regions, including HMEL014795g1 and HMEL015842g1 (at 10Mb and ~13Mb on chromosome 18, respectively) and HMEL030024g1 (on chromosome 1) might be considered good candidates for *trans*-regulation. However, in our backcrosses, the region on chromosome 18 introgressed from *H. melpomene* into a *H.* cydno background extends ~3.6Mb beyond the QTL candidate region, making it difficult to determine whether these genes are regulated by loci associated with variation in behaviour."

Perhaps this goes beyond the reviewers intended point, but we have now also added a paragraph in the Supplement at L89-97 about the possibility of a cis-regulatory element within the QTL acting over long-distance (regulating a gene outside the QTL). More generally however, the average distance between regulatory elements and a gene seems to be less than 100kb (Gaffney 2019 *Nat. Gen.),* compared to 2.75 Mb of the candidate QTL region. Overall, we think that focusing on genes within the QTL region is a valid approach to identify the most likely candidates.

In general, the compactness of the manuscript means a few details are hard to follow. It wasn't clear to me if the two candidate genes were differentially expressed across all developmental stages (it is hard to distinguish individual genes in Figure 2).

We have now made this information more explicit in the Figure 2 legend:

"*Grik2* and *regucalcin2* at the adult stage, Grik2 at 60h APF",

and we have also specified this at L195-196

"*Grik2* was the only gene differentially expressed between species and hybrids at these pupal stages (at 60h APF, Supplementary figure 4)."

How does this relate to the BC where only pupal stages were sequenced? The results should clarify which BC samples are being compared (which is not presented until the methods, Lines 456-459).

In the results (L188-191) we now state:

"We compared 6 *cyd/melp* vs. 10 *cyd/cyd* (at the QTL region on chromosome 18) BC3 hybrids sampled at 156 hours after pupal formation (APF) (Supplementary figure 5A), and 8 *cyd/melp* vs. 9 *cyd/cyd* for those at 60h APF.*"*

After three generations of backcrossing, what was the average genome composition (outside of Chr 18, Lines 454-455)? I indicated other details that might be helpful in my comments below.

We have added this estimate at L527-530:

"The average percentage of genome that is heterozygous (*cyd/melp)* as opposed to homozygous (*cyd/cyd)* outside of chromosome 18 was ~6% (close to the expectation that a 3rd generation backcross genome should be 1/16 heterozygous (*cyd/melp*))"

While the ASE analyses provide some support for the cis regulatory change, they are not particularly conclusive. How low were the number of reads that mapped to diagnostic SNPs and was that read mapping sufficient for the analyses (this might require a power analysis?). ASEReadCounter filters out duplicate reads by default, and it looks like the default settings were used for this analysis (Line 471). This is a strange default setting given that RNAseq datasets typically have many reads that are identical due to real expression differences. Could this have impacted the number of reads for the ASE mapping?

We are very grateful to the reviewer for pointing out that ASEReadCounter deduplicates reads by default, which was an oversight on our part (and we agree that this is not optimal for RNA-seq reads). We have now redone these analyses without deduplication (*i.e.* with option -drf DuplicateRead). The results are effectively the same as our previous analysis for both the F1 hybrids (only a handful of allele-informative reads were rescued) and backcrosses, with the exception of regucalcin2 at one pupal stage (60h APF) where we now have evidence for cis-regulatory effects.

If there are insufficient reads in the total RNAseq dataset, why not use targeted sequencing of these genes to estimate ASE?

We agree that the number of available reads for our previous ASE analysis was somewhat unsatisfying. We have redone these analyses but including 6 new F1 male hybrid samples (which were recently collected for another project). As a result, we

now have more power to demonstrate that both genes show evidence of cis-regulatory effects.

Considering other regulatory categories may also be helpful (e.g., cis + trans, cis x trans, compensatory) given the differences in cis vs trans changes between adult and pupal stages. I recognize these cutoffs are fuzzy and somewhat arbitrary, but if I am looking at Figure 3 correctly, it looks like Grik2 may be cis+trans given that the mel allele has higher expression in the parents than in than in the hybrids. At the very least, I think it is worth noting that with low sequencing depth, whether Grik2 is cis/trans or both at different stages is not exactly clear.

We agree that the effects, particularly for Grik2, seem mixed (not just the effect of *cis*-regulation). We now address this in the Figure 3 legend:

"Both genes seem to be regulated by a combination of cis- *and* trans-acting factors, rather than cis-acting factors alone (which would be indicated by y=x)".

Nevertheless, we think that expanding on this further would introduce an extra level of detail that is not too important for the results, as there need only be a change in cis-regulation for either gene to be causal.

Minor Comments

Line 36-37: To be fair, the male calling song and female preference in crickets has now been mapped in a QTL study with a very promising candidate gene, a nucleotide-gated ion channel gene (Xu and Shaw, 2019, Genetics). This study should be cited in this paragraph.

This was an oversight, we have now added this citation.

Line 58: It would be nice to integrate the sexual selection literature here – signals and preferences are predicted to be linked.

Perhaps we are not interpreting the reviewer's point correctly, but our understanding is that linkage disequilibrium (rather than physical linkage *per se*) is predicted to underpin trait-preference correlations (and therefore Fisher processes), and in this case LD comes about behaviourally (*i.e.* as a result of preferences) (see for example Bakker & Pomiankowski 1995 JEB; Hosken & Wilson 2019 Behavioural Ecology). It seems possible to us that physical linkage would facilitate Fisherian runaway selection, and this scenario seems likely to affect speciation in systems like *Laupala* for example; however, we know of no specific study that addresses this – any suggestions from the reviewer would be welcome. This is in contrast to the speciation literature where it is well established that physical linkage between (ecological) traits under divergent selection and those contributing to pre-mating isolation will facilitate speciation (and for this reason we cite Felsenstein 1981 *Evolution*, where we think this was first made explicit). In addition, we cite two recent reviews in the discussion at Line 261-262 (Kopp et al. 2018 Am. Nat., Butlin & Smadja 2018 Am. Nat), where these ideas are discussed.

Line 86: It might be worth mentioning here that collectively these results suggest the mel allele is dominant, since this is referred to later in the manuscript (Line 124).

We have now elaborated as follows:

"[...] these results suggest that the QTL on chromosome 18 harbours genes for visual attraction behaviours towards females with the red pattern (and that the *H. melpomene* alleles are dominant)."

Line 93: Supplemental Table 1 A reports DE results for Chr 1, so why is this cited here for QTL analysis?

We have moved this sentence to the next paragraph, now at L128-129.

Line 100: Maybe change imago to adult to avoid unnecessary entomology jargon?

Changed.

Line 106: How many genes were DE across the rest of the genome, and are there more/less DE genes on Chr 18 than expected compared to other autosomes?

The number of all DE genes across the rest of the genome is 1354, 1490 and 1473 at Adult, 156h APF and 60hAPF stages respectively. Please see our response below, which is the same as to reviewer 2, who asked a similar question:

Our intention here was not to suggest that gene expression is higher in this region compared to other parts of the genome, but simply that there were a number of differentially expressed genes. To avoid this confusion, we have changed these sentences so that they now read:

"Across the QTL region on chromosome 18 (which spans 2.75 Mb, and contains 200 genes), we identified 27 genes within that show differential expression between *H. melpomene* and *H. cydno*, in at least one of the three developmental stages."

Chromosome 18 is not enriched for differentially expressed genes (Fisher's Exact test: $P > 0.05$, for all three stages), though we don't think this is relevant for our results (and would expect a large number of differentially expressed genes across the genome).

Line 114: It is a bit weird to refer to mapping bias as from divergent coding sequences. any SNPs can cause mapping bias?

We thank the reviewer for pointing this out and we agree that for example UTRs might influence RNAseq reads mapping, so we have changed "coding" sequences into divergent "gene" sequences.

Lines 115-116 and Line 43 in Supplement: Could the manuscript include the number of reads mapped to each reference for each sample?

We have now added Table S2B to the Supplement, indicating the number of reads that mapped to the *H. melpomene* genome and to the *H. cydno* genome for each species sample at the adult stage. Mapping rates to the two genomes are different, and less reads map to the *H. cydno* genome (and less genes are detected as differentially expressed when mapping to the *H. cydno* genome, which likely reflects the lesser quality of the *H. cydno* genome assembly/annotation as pointed out at L328-329 of the Supplement).

Nevertheless, we think Table S2A effectively shows that there is no bias in the inference of up-/down-regulation based on the reference genome: For example, it is not the case that the proportion of genes up-regulated in *H. melpomene* is higher when mapping to *H. melpomene* (or up-regulated in *H. cydno* when mapping to *H. cydno*), which might have otherwise suggested a bias in the inference of differential expression based on the reference genome (which was our concern).

Line 128: There were no genes on Chr 1 that were DE between F1 hybrids and cydno?

There were 2 genes, one with no annotated function and a gene annotated as "Regulation of enolase protein 1", we have added this information to the Supplementary table 1A.

Line 140: It could be worth clarifying here that the expectation is that a DE gene within the QTL interval would be regulated via cis change if that DE gene is associated with the QTL.

We have added this sentence at L165-170: "Causal changes in gene regulation underlying phenotypic variation associated with QTL must result from *cis*- rather than *trans*-regulation. In other words, if changes in expression of *Grik2* and *regucalcin2* account for the observed shifts in behaviour associated with the QTL these must be due to changes within the *cis*-regulatory regions of the genes themselves (as opposed to of other *trans*-acting genes elsewhere in the genome); and these causal mutations must be within our QTL region on chromosome 18.).

Line 163: Was DE analysis for the BC done only on pupal stages (i.e. not adults)?

Generating backcrosses across multiple generations is rather difficult in *Heliconius*, and we were limited by the number of hybrids produced (and the number of samples we could sequence). When the sequencing experiment was planned the expectation was that the genetic change would exert its action during pupal development (e.g. visual circuit assembly), so we decided to focus the sequencing effort on those pupal stages, although we acknowledge that not having sampled backcrosses at the adult stage is a caveat of this study.

Line 348: Perhaps clearer to keep referring to this introgression line as a backcross?

We agree with the reviewer that this makes things clearer, we have changed "The introgression line was generated by [...]" into "Third-generation backcross hybrids (BC3) were generated by [...]"

Line 392: There are differences in the cell composition/structure of the brains between species? Perhaps mention this in the discussion?

We have now updated the reference "Montgomery et al. in prep" at L458 to the actual preprint that shows that neuroanatomical differences between *H. melpomene* and *H. cydno* are clustered in the visual system. We have expanded on this point briefly, but do not think it renders extensive discussion as there is little expectation that visual mating preference is linked to these divergent brain morphologies. Furthermore, Montgomery *et al* also report neuroanatomical differences (in the visual systems) of *H. melpomene* (*amaryllis*) and *H. timareta* (*thelxinoe*), but given that they are (red) co-mimics and neither appear to distinguish conspecifics using visual cues (Mérot et al. 2015, Evolution), the shift in visual investment is unlikely to be related to mate choice.

Instead, the neuroanatomy of *H. timareta* is more similar to that of *H. cydno* to which it is more closely related, and with which it shares a more similar ecology (as compared to *H. melpomene).*

From a methodological perspective, including a strong fold-change threshold for detecting shifts in gene expression counters possible influence of changes in tissue composition on our results. L456-458 now read:

"[...] to exclude expression differences caused by known differences in brain morphology[75], that in this species pair are clustered in the visual system[76]"

Figure 2: There is a fuchsia vertical line indicating a gene that was excluded because it did not show differential expression or showed the reversal fold change when mapping to the cydno genome. Given the proximity of this gene to the DE genes identified as candidates, could this be clarified? Is this because there was less power to identify candidate genes with the cydno genome? Or is it because there is divergence or structural reasons why reads are mapping differently in that region?

The reversal of the fold change when mapping to the *H. cydno* genome for that gene (HMEL034187g1) is due to the incompleteness of its annotation: it doesn't include exons that seem to be part of it (based on spliced-reads coverage). As a result, a considerable part of this gene is composed by the UTR region, which is highly divergent, leading to a reversal of the fold change when mapping to the two genomes.

Using the transcript-guided annotation (where this gene's annotation now includes those other exons) HMEL034187g1 is no longer differentially expressed. We have now added the following to the Results at Line 149-155 (which previously was stated only in the method section):

"To ensure that apparent fragmentation of a few gene models in the *H. melpomene* (Hmel2.5) annotation[32] did not introduce inaccuracies in estimates of differential gene expression, we produced a new transcript-based annotation of the *melpomene* genome using Cufflinks's RABT[33]. Repeating all comparative transcriptomic analyses using this new annotation (where exons previously considered to be distinct genes, are now assigned correctly to single genes), we confirmed that both re*gucalcin2* and *Grik2* were differentially expressed in both species and hybrids comparisons."

In general, there seems to be less power to identify genes in the *H. cydno* genome, which is most likely due to the lower quality of its assembly (the number of gene models annotated in *H. melpomene* that then were identified in *H. cydno* was 17478/19609, Darragh et al. biorXiv), but our priority when including these analyses (mapping to *H. cydno*) was to exclude artefacts of the reference genome on our inferences of differential expression.

REVIEWERS' COMMENTS:

Reviewer #2 (Remarks to the Author):

The authors answered most of my previous comments, but some corrections are still needed. I think their results bring strong evidence for the implication of the QTL peak located on chromosome 18 in explaining variations in visual preferences, but the QTL 1 also seem to play a significant role and is too rapidly discarded from the subsequent analyses. The Grik2 and regulcalcin genes are indeed strong candidates, but some alternative candidates might also be important and the lack of functional analysis prevents formal conclusions. The discussion provides a more nuanced view, by showing the alternative hypothesis on the visual vs. neuronal components of visual preferences evolution and highlighting that the results are in line with the second hypothesis. This should be reflected in the abstract and title: the implication of neural processes in the evolution of visual preference is supported by the results but the causative gene(s) involved in the behavioural switch are not fully confirmed.

Line 14-15 : This sentence is still not correct and should be entirely rephrased, species do not produce offspring, they do not fail or succeed. This is important, because these erroneous shortcuts do not reflect the actual speciation process.

Line 53: Similarly, a species does not mimic another species, but colour pattern of H. cydno are mimetic of wing coloration of H. sapho. This should be properly written.

Line 63: In my opinion, this is a working hypothesis and should be presented as such. You may thus correct this sentence by adding : 'SUCH genetic linkage IS EXPECTED TO facilitate speciation by impeding the breakdown of genetic associations between ecological and mating traits'.

Line 99-101: I am still not convinced that it is relevant to ignore the effect of the QTL located on chromosome 1 when studying the effect of the QTL located on chromosome 18. Supplementary material confirms the effect of genotype at chromosome 1 on visual preference and potential interaction with genotype at chromosome 18. A more exhaustive study would allow drawing firmer conclusion on the significance of neural processing genes in the evolution of visual preferences.

Line 128-129: This sentence should summarize whether or not differentially expressed genes were also detected in the region of QTL located on chromosome 1, and if so whether genes involved in neural processing were identified. As mentioned in the supplementary material, the QTL peak on chromosome 1 also contains differentially expressed genes and there is no reason to discard these genes from the list of interesting candidate genes.

Line 251-254: As I mentioned in my previous review, the whole genomic region of the QTL peak (in green on figure 4) has a low fd, but the regulcalcin 1 and 2 are not specially associated with a reduced fd. I am thus not convinced that the fd pattern strengthen the relevance of the candidate genes identified by the QTL analysis. As mentioned in your answer, regulatory regions of these genes can be located further away, so I am not sure about the relevance of the figure 4 as confirming the divergent selection on the candidate genes.

Reviewer #3 (Remarks to the Author):

I think the revised manuscript is very well done and the authors have been very thorough in responding to my questions. They have even added additional data that strengthens their results. I have no further questions about the manuscript, though I do respond to a few of the authors comments below:

Since writing my review, I have learned that some ASE pipelines do recommend removing duplicate reads (see Castel et al. Genome Biology, 2015), but it was good to check the settings and find consistent results either way.

In regards, to the sexual selection literature, yes, I was thinking of evidence from Laupala and this paper does an excellent job of outlining the idea that signals and preference by the receiver may be linked (Wiley, Ellison, Shaw. 2012. Proc. R. Soc. B). There are also these older reviews (Butlin & Ritchie 1989 Biol. J. Linn. Soc., and Boake 1991 Trends Ecol. Evol). As the authors point out, color pattern in this system is likely influenced by both sexual and ecological selection, so I agree, this is an aside and probably isn't worth mentioning.

REVIEWERS' COMMENTS:

Reviewer #2 (Remarks to the Author):

The authors answered most of my previous comments, but some corrections are still needed. I think their results bring strong evidence for the implication of the QTL peak located on chromosome 18 in explaining variations in visual preferences, but the QTL 1 also seem to play a significant role and is too rapidly discarded from the subsequent analyses. The Grik2 and regulcalcin genes are indeed strong candidates, but some alternative candidates might also be important and the lack of functional analysis prevents formal conclusions. The discussion provides a more nuanced view, by showing the alternative hypothesis on the visual vs. neuronal components of visual preferences evolution and highlighting that the results are in line with the second hypothesis. This should be reflected in the abstract and title: the implication of neural processes in the evolution of visual preference is supported by the results but the causative gene(s) involved in the behavioural switch are not fully confirmed.

We thank the reviewer for their useful comments. With respect to the abstract, it is of course a very short summary of our findings: Nevertheless, we note that we refer to '*candidates*' which '*suggest* shifts in behaviour involve changes in visual integration or processing', which we think is appropriate and in line with the discussion.

Line 14-15: This sentence is still not correct and should be entirely rephrased, species do not produce offspring, they do not fail or succeed. This is important, because these erroneous shortcuts do not reflect the actual speciation process.

We have rephrased the sentence, so as not to give the impression that species produce offspring:

"Many animal species remain separate not because their individuals fail to produce viable hybrids but because they "choose" not to mate."

However, we disagree that 'fail' necessarily implies intention, after all bridges fail, pregnancies fail etc. etc.

Line 53: Similarly, a species does not mimic another species, but colour pattern of H. cydno are mimetic of wing coloration of H. sapho. This should be properly written.

We have changed the sentence so that it now reads:

"*In central Panama, *H. melpomene* shares the black, red and yellow pattern of its local *Heliconius erato* co-mimic, whereas *H. cydno* shares the black and white patterns of *H. sapho*.*"

Line 63: In my opinion, this is a working hypothesis and should be presented as such. You may thus correct this sentence by adding: ‘SUCH genetic linkage IS EXPECTED TO facilitate speciation by impeding the breakdown of genetic associations between ecological and mating traits’.

We have changed “will facilitate” to “is expected to facilitate”.

Line 99-101: I am still not convinced that it is relevant to ignore the effect of the QTL located on chromosome 1 when studying the effect of the QTL located on chromosome 18. Supplementary material confirms the effect of genotype at chromosome 1 on visual preference and potential interaction with genotype at chromosome 18. A more exhaustive study would allow drawing firmer conclusion on the significance of neural processing genes in the evolution of visual preferences.

We assume the reviewer is referring to the analysis of the backcrosses, and we agree in principle that any epistasis between these loci might be important for interpreting our results. However, we did not find evidence of an interaction between the QTL on chromosome 1 and chromosome 18 in our previous analyses (Merrill et. al 2019, *PLoS Biol*.); We have also checked for an interaction in our current analysis of courtship initiation and again find little evidence. To clarify this important point, we have added the following sentence to L99-101:

“As in our previous analyses of relative courtship time[20], we found little evidence for an interaction between the QTLs on chromosome 1 and 18 (including the interaction led to a worse fit compared to a model excluding the interaction: ΔELPD: -1.7 (S.E.±0.9), *i.e.* a change of 1.89 SE units), suggesting additive effects.”

Line 128-129: This sentence should summarize whether or not differentially expressed genes were also detected in the region of QTL located on chromosome 1, and if so whether genes involved in neural processing were identified. As mentioned in the supplementary material, the QTL peak on chromosome 1 also contains differentially expressed genes and there is no reason to discard these genes from the list of interesting candidate genes.

Although our focus is explicitly on the QTL on chromosome 18, we agree this is potentially important. We have now clarified these results (L 98-101):

“Although our focus was primarily on chromosome 18, two genes within the QTL region on chromosome 1 were also differentially expressed for at least one developmental stage (Supplementary table 1), when mapping to the *H. melpomene* and *H. cydno* genomes. Of these, one (HMEL003796g1), associated with the regulation of enolase, was also upregulated in F1 hybrids (see below).”

Line 251-254: As I mentioned in my previous review, the whole genomic region of the QTL peak (in green on figure 4) has a low fd, but the regulcalcin 1 and 2 are not

specially associated with a reduced fd. I am thus not convinced that the fd pattern strengthen the relevance of the candidate genes identified by the QTL analysis. As mentioned in your answer, regulatory regions of these genes can be located further away, so I am not sure about the relevance of the figure 4 as confirming the divergent selection on the candidate genes.

We maintain that the results are consistent with divergent selection on candidate genes. Although we agree that the *regucalcins* are not associated with the lowest possible f_d values at their exact location, the window-based estimation of f_d makes it subject to variability stemming from nearby regions/genes, which might have been subjected to opposing evolutionary forces (especially since the species are at the later stage of divergence). It is difficult to delineate the exact source of this variability, but we maintain that since the regions around the *regucalcins* show reduced levels of f_d, this is still consistent with divergent selection acting on our candidate genes (especially for regucalcin 2, for which regulatory regions might be, perhaps, 50kb away, coinciding with extremely reduced f_d). Although we concede that interpreting these kinds of genomic data is difficult, we believe it is an important additional piece of evidence.

Reviewer #3 (Remarks to the Author):

I think the revised manuscript is very well done and the authors have been very thorough in responding to my questions. They have even added additional data that strengthens their results. I have no further questions about the manuscript, though I do respond to a few of the authors comments below:

Since writing my review, I have learned that some ASE pipelines do recommend removing duplicate reads (see Castel et al. Genome Biology, 2015), but it was good to check the settings and find consistent results either way.

In regards, to the sexual selection literature, yes, I was thinking of evidence from Laupala and this paper does an excellent job of outlining the idea that signals and preference by the receiver may be linked (Wiley, Ellison, Shaw. 2012. Proc. R. Soc. B). There are also these older reviews (Butlin & Ritchie 1989 Biol. J. Linn. Soc., and Boake 1991 Trends Ecol. Evol). As the authors point out, color pattern in this system is likely influenced by both sexual and ecological selection, so I agree, this is an aside and probably isn’t worth mentioning.

We thank the reviewer for the positive comments and for pointing us towards these useful studies on ASE pipelines and linkage of signals and preferences. We are also very grateful for their previous comments which we think greatly improved the paper.
